# The study of *Priapulus caudatus* reveals conserved molecular patterning underlying different gut morphogenesis in the Ecdysozoa

**DOI:** 10.1186/s12915-015-0139-z

**Published:** 2015-04-21

**Authors:** José M Martín-Durán, Andreas Hejnol

**Affiliations:** Sars International Centre for Marine Molecular Biology, University of Bergen, Thormøhlensgate 55, 5008 Bergen, Norway

**Keywords:** *C. elegans*, *Drosophila*, Ecdysozoa, Endoderm, Gut development, Hindgut, Mesoderm, Midgut, Mouth, Priapulid

## Abstract

**Background:**

The digestive systems of animals can become highly specialized in response to their exploration and occupation of new ecological niches. Although studies on different animals have revealed commonalities in gut formation, the model systems *Caenorhabditis elegans* and *Drosophila melanogaster*, which belong to the invertebrate group Ecdysozoa, exhibit remarkable deviations in how their intestines develop. Their morphological and developmental idiosyncrasies have hindered reconstructions of ancestral gut characters for the Ecdysozoa, and limit comparisons with vertebrate models. In this respect, the phylogenetic position, and slow evolving morphological and molecular characters of marine priapulid worms advance them as a key group to decipher evolutionary events that occurred in the lineages leading to *C. elegans* and *D. melanogaster*.

**Results:**

In the priapulid *Priapulus caudatus*, the gut consists of an ectodermal foregut and anus, and a mid region of at least partial endodermal origin. The inner gut develops into a 16-cell primordium devoid of visceral musculature, arranged in three mid tetrads and two posterior duplets. The mouth invaginates ventrally and shifts to a terminal anterior position as the ventral anterior ectoderm differentially proliferates. Contraction of the musculature occurs as the head region retracts into the trunk and resolves the definitive larval body plan. Despite obvious developmental differences with *C. elegans* and *D. melanogaster*, the expression in *P. caudatus* of the gut-related candidate genes *NK2.1*, *foxQ2*, *FGF8/17/18*, *GATA456*, *HNF4*, *wnt1*, and *evx* demonstrate three distinct evolutionarily conserved molecular profiles that correlate with morphologically identified sub-regions of the gut.

**Conclusions:**

The comparative analysis of priapulid development suggests that a midgut formed by a single endodermal population of vegetal cells, a ventral mouth, and the blastoporal origin of the anus are ancestral features in the Ecdysozoa. Our molecular data on *P. caudatus* reveal a conserved ecdysozoan gut-patterning program and demonstrates that extreme morphological divergence has not been accompanied by major molecular innovations in transcriptional regulators during digestive system evolution in the Ecdysozoa. Our data help us understand the origins of the ecdysozoan body plan, including those of *C. elegans* and *D. melanogaster*, and this is critical for comparisons between these two prominent model systems and their vertebrate counterparts.

**Electronic supplementary material:**

The online version of this article (doi:10.1186/s12915-015-0139-z) contains supplementary material, which is available to authorized users.

## Background

A defining character of animals is the need to incorporate other organisms, or their products, for nourishment. Although different strategies have evolved to accomplish this task [1,2], the solution present in almost all metazoans is the development of organs with specialized cell types to ingest and digest food, and absorb the resulting nutrients. The digestive system is thus a central morphological and physiological constituent of metazoans, and, as such, has experienced intense adaptation and diversification, as animals have radiated into different ecological niches and utilized new food sources and predatory strategies [1]. Accordingly, how this variety of digestive systems originated emerges as a key question in the study of animal body plan evolution.

Whereas many early-branching animal lineages, such as Cnidaria (that is, jellyfish, corals), show a sack-like intestine that opens to the exterior through the mouth, most bilaterally symmetrical animals (for example, mammals, flies, and earthworms) exhibit a through gut with two openings, the mouth and the anus, and distinct regions specialized for particular feeding tasks [1]. Pharynxes, jaws, and proboscides to capture and grind food, stomachs and digestive glands to process nutrients, and cloacae to release excretory products are just a few examples of the specializations exhibited by animal digestive systems. Despite this diversity in gut architecture and complexity, the comparative study of different bilaterian animals has revealed commonalities in the early ontogenetic stages of gut formation, and a handful of genes have been related to the specification and initial development of the digestive system [3-6]. The gut usually forms from a population of cells that are localized at one point of the early embryo and that get internalized in a process called gastrulation [7]. These cells, the endoderm (literally, internal skin) of the embryo, form the most medial part of the intestine, which opens into the ectoderm (external skin) through the mouth and the anus. Beyond these broad commonalities, the way in which the gut forms may significantly change as organisms undergo developmental adaption in response to *de novo* habitat colonization [8-10].

**Figure 1:**
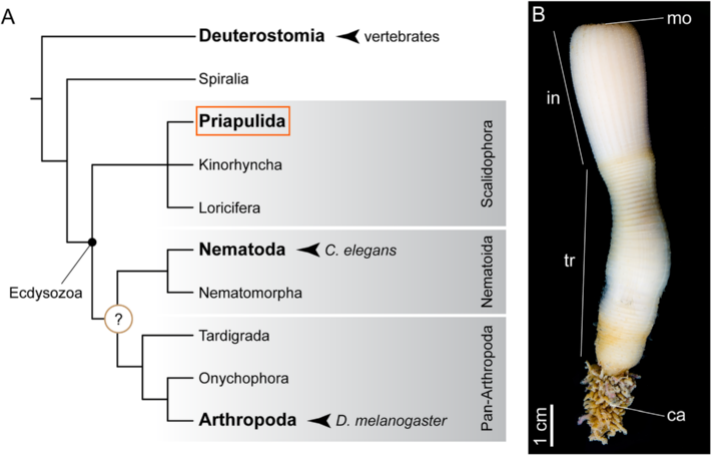
Ecdysozoan model systems and the reconstruction of ancestral characters. **(A)** The Ecdysozoa (molting animals) comprises three main lineages, namely Scalidophora, Nematoida, and Pan-Arthropoda. *C. elegans* and *D. melanogaster*, the two most important invertebrate model systems, belong to Nematoida and Pan-Arthropoda, respectively. The study of a representative of the third main ecdysozoan lineage, the Scalidophora, and in particular of the marine Priapulida, will shed light on ancestral character states present at the base of the Ecdysozoa, and thus on the evolutionary events that occurred in the lineages leading to nematodes and arthropods. Phylogenetic relationships are according to [29]. **(B)** Adult priapulid of the species *Priapulus caudatus*. Priapulids are sausage-shaped annulated worms, with an anterior introvert (in), a terminal mouth (mo), a trunk (tr), and a posterior caudal appendage (ca).

Two extremely specialized modes of gut development are observed in the terrestrial nematode *Caenorhabditis elegans* and the fruit fly *Drosophila melanogaster*, the most widely used invertebrate model systems in developmental biology and biomedical research [11-13]. Both the nematode and the fruit fly belong to the Ecdysozoa (molting animals) [14] (Figure 1A), which is one of the three main animal lineages that form the Protostomia, together with the Spiralia and the enigmatic Chaetognatha [15-17]. In *C. elegans*, the entire tube-like intestine consists of 20 cells; it opens anteriorly through a buccal cavity and a muscular pharynx of less than 100 cells, and posteriorly through an ectodermal hindgut of 11 cells [18-20]. The 20 intestinal cells are clonal, and originate from a single founder cell, the E blastomere, at the eight-cell embryo [21-24]. The E cell first divides once on the surface of the embryo, and the resulting daughter cells migrate into the embryo during gastrulation. Three rounds of cell division generate a 16-cell intestinal primordium, in which cell differentiation and lumen formation take place [18]. *C. elegans* is thus the archetypal example of an animal with a highly stereotypic determinative development, reduced number of cells, and fast life cycle [21]. Differing from *C. elegans*, the development of *D. melanogaster*, as also observed in many other arthropods, is strongly adapted to terrestrial environments and yolky eggs [10]. The more complex digestive system of the fly *D. melanogaster* is made of thousands of cells, and is divided into an ectodermal foregut (mouth, esophagus, crop, and proventriculus), an endodermal midgut subdivided into at least six physiological regions, and an ectodermal hindgut [25]. Strikingly different from most animals, the endoderm is specified in two different regions of the embryo before gastrulation, namely the anterior and the posterior midgut primordia [26]. These two cell populations undergo an epithelial to mesenchymal transition, and ingress inside the embryo, which is filled by yolk. The foregut and hindgut, which surround the anterior and posterior midgut primordia respectively, invaginate after the mesenchymal endodermal cells. The two endodermal populations then migrate through the embryo to eventually meet at the middle, and re-epithelialize to define the digestive tract [27], in a process tightly coupled with the development of the visceral mesoderm [28]. Although *C. elegans* and *D. melanogaster* are by far the two best-studied ecdysozoans, their highly peculiar and adaptive modes of development hinder the reconstruction of ancestral and derived characters for nematodes and arthropods (Figure 1A). Therefore, alternative taxa are needed to understand the evolutionary origins of the development of the digestive tract in these two model systems and in the Ecdysozoa as a whole, which is ultimately essential for the interpretation and translation of the research conducted on *C. elegans* and *D. melanogaster* to model vertebrate systems, such as the frog, fish and mouse.

Most recent phylogenies place the exclusively marine priapulid worms (Priapulida), and the related taxa kinorhynchs and (likely) loriciferans, as the earliest branching ecdysozoan lineage (Scalidophora), and thus the sister group to the remaining ecdysozoans, including nematodes and arthropods [15,17,29] (Figure 1A). The extant Priapulida comprise only 19 described species [2,30], but were among the most abundant and widespread animals in the Early Cambrian [31]. The oldest trace fossils from the beginning of the Cambrian (*Treptichnus pedum*) resemble burrowing priapulids, or morphologically very similar animals [32]. Priapulids, commonly referred to as penis worms, are large sized (0.5 to 20 cm), mud-dwelling or interstitial annulated worms, with an anterior proboscis (or introvert), and a terminal mouth [2,33] (Figure 1B). Reports on their embryonic development are scarce and mostly focused on the early stages of development of the species *Priapulus caudatus* Lamarck 1816 [34,35]. *P. caudatus* reproduces by external fertilization, and the small embryos undergo holoblastic radial cleavage, gastrulation by invagination and epiboly [35], and deuterostomic formation of the mouth [34], which are all considered to be plesiomorphic features in the Ecdysozoa [34,36]. This combination of characters, together with their slow rate of molecular evolution [37], render the Priapulida, and in particular the representative species *P. caudatus*, as the key conservatively evolving ecdysozoan group to compare with nematodes and arthropods, and to thereby infer ancestral characters for these species-rich lineages of animals.

In the present study, our aim was to characterize the formation of the gut in *P. caudatus* and then, by comparing our data with the knowledge on *C. elegans*, *D. melanogaster*, and other bilaterians, to decipher the evolutionary events that occurred after cladogenesis of the nematode and arthropod lineages. Principally, we focused on the morphological development of the endoderm into the definitive intestine, as well as on how the mesoderm segregates from the endoderm and its putative influence on the formation of the gut. We then analyzed mouth and head development, as well as the molecular regionalization of the definitive digestive system, by studying the expression of the mouth markers *NK2.1*, *foxQ2*, and *FGF8/17/18*; the midgut markers *GATA456* and *hepatocyte nuclear factor 4* (*HNF4*); and the hindgut markers *wnt1* and *even-skipped* (*evx*). Our data shed light on the origins and evolution of the digestive tracts of *C. elegans*, *D. melanogaster*, and the Ecdysozoa in general. Importantly, our data demonstrate that a conserved molecular patterning system underlies the great variability of ontogenetic modes and architectures observed in the digestive systems of ecdysozoans.

## Results

### Gut formation in *P. caudatus*

**Figure 2:**
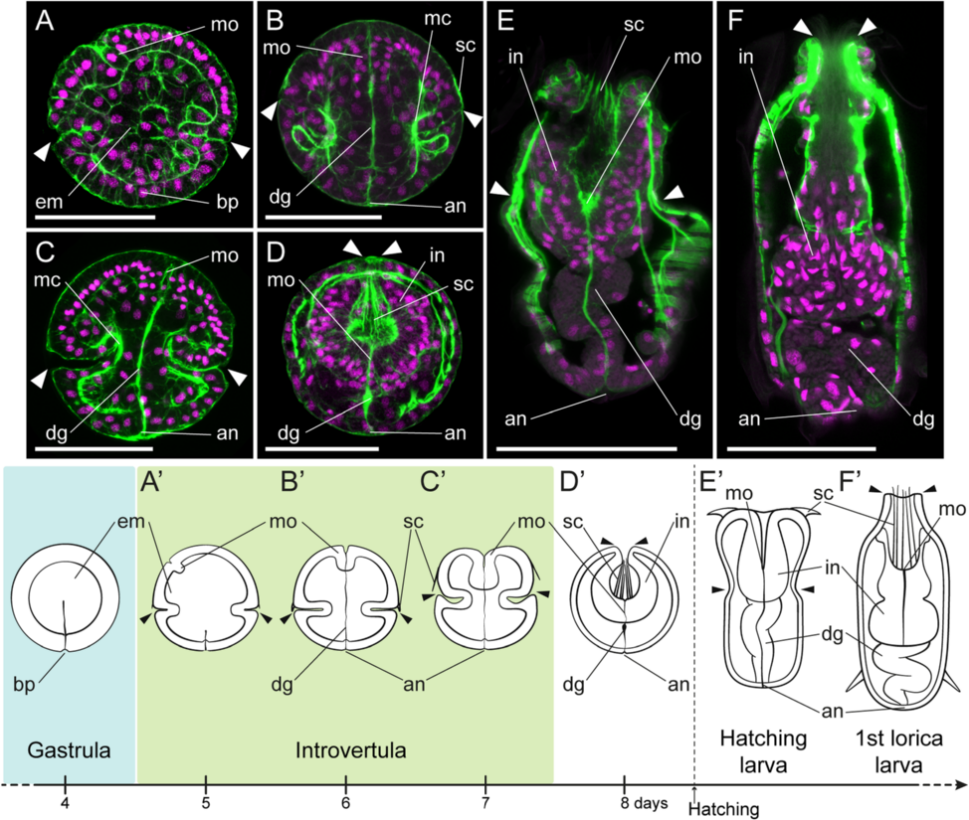
Gut formation during *P. caudatus* embryogenesis. z projections of confocal stacks of embryos at 5, 6, 7, and 8 days post-fertilization, hatching larva, and the first lorica larva, stained with phallacidin (green) and propidium iodide (magenta). **(A)** Post-gastrula embryos exhibit a parenchymatous endomesoderm (em), a ventrally forming mouth (mo), and a narrowed blastopore (bp) that will give rise to the anus. **(B)** With the onset of organogenesis, the mouth moves to an anterior terminal position, and a digestive tract (dg) connecting the mouth and the anus (an) is visible as a strongly actin-positive bundle. Muscle (mc) differentiation also starts at this stage. **(C,** **D)** After organogenesis, the introvert retracts into the trunk, pulling down the digestive tract and mouth to the posterior end of the trunk. The scalids (sc), which develop at the introvert-trunk boundary **(B, B’)**, are located at the anterior end of the introvert as it retracts. **(E)** The hatching larva exhibits a fully developed digestive system, despite the lack of a mouth and anal opening in the cuticle. **(F)** With the first molting, the first lorica larva exhibit greater body complexity, and the digestive system increases its number of cells generally. **(A’-F’)** Schematic drawings of the studied embryonic and larval stages, placed in the general context of priapulid embryonic development. The pairs of arrowheads indicate the position of the introvert-trunk boundary. Drawings are not to scale. All panels and drawings are oriented with the animal/anterior pole to the top. In **A**, ventral side to the left. Scale bars in **A-D** 50 μm; and **E** and **F**, 100 μm.

Gastrulation is usually the first morphogenetic step in the formation of the digestive tract in metazoans [7,38]. During this event, the endomesodermal cells, which will form the digestive system and mesodermal derivatives, internalize and segregate from the external ectoderm. In *P. caudatus*, gastrulation occurs at the vegetal pole [35], and after endomesoderm ingression, the embryo shows an obliterated archenteron, and a narrowed blastopore that corresponds to the future anal opening of the digestive tract [34]. At this stage, between days 4 and 5 of development, the endomesoderm exhibits a parenchymatic appearance (Figure 2A), without any obvious morphological differences between the future endodermal cells and the mesoderm. The mouth appears ventrally in the animal hemisphere [34], as an ectodermal invagination of a few cells (Figure 2A, A’), and a subequatorial ectodermal groove marks the division of the body into an anterior/animal introvert and a posterior/vegetal trunk (introvertula stage).

**Figure 3:**
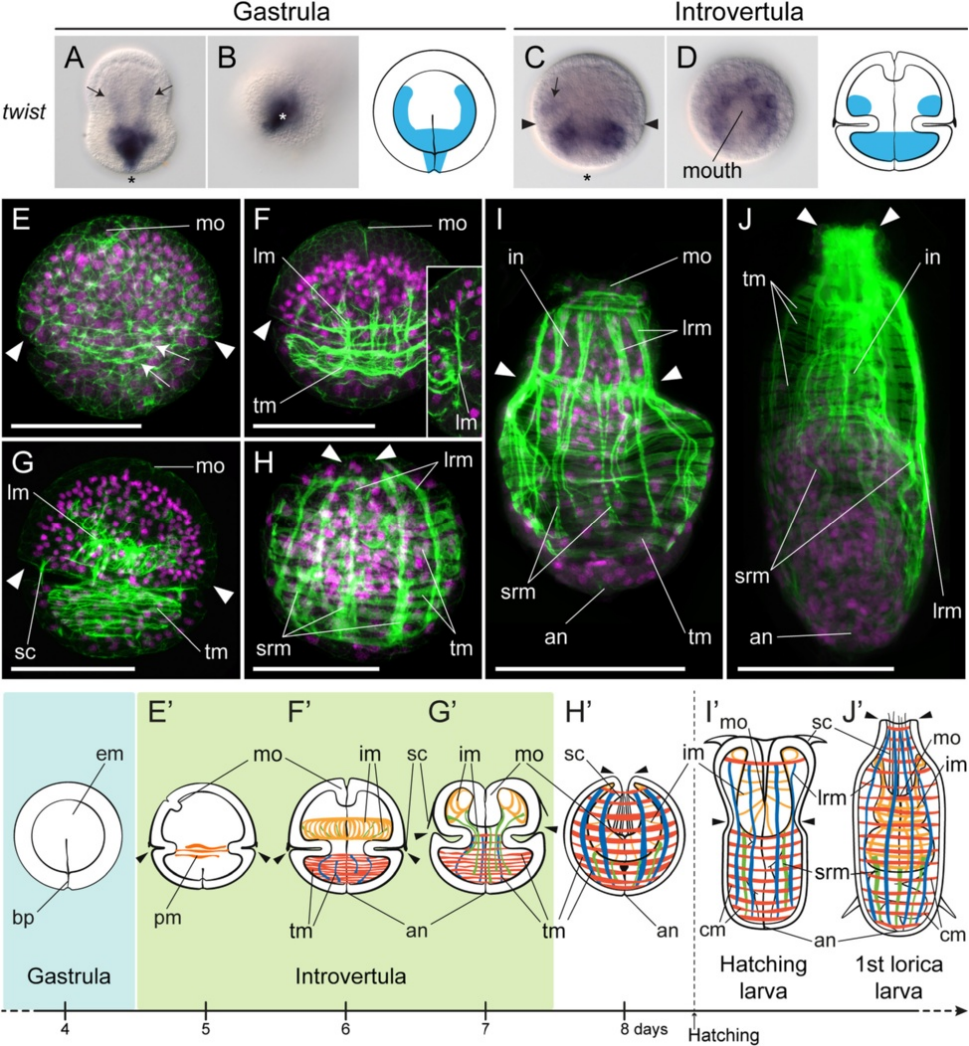
Mesoderm specification and myogenesis in *Priapulus caudatus*. **(A-D)** Whole-mount *in situ* hybridization of *twist. twi* is expressed in the most vegetal endomesodermal cells, and in two lateral bands of the gastrula (black arrows in **A**) and later in the developing musculature of the introvert (black arrow in **C**) and trunk. In **(A-C)** the asterisk indicates the blastopore/anus. Drawings depict the expression domains. The slight elongation of embryos might be an artifact of fixation. **(E, F)** z projections of confocal stacks of embryos at 5, 6, 7, and 8 days post-fertilization, hatching larva, and the first lorica larva, stained with phallacidin (green) and propidium iodide (magenta). **(E)** Muscle fibers appear as actin-positive bundles around the equator of the embryo (white arrows). **(F)** Subsequently, the trunk musculature and longitudinal muscles (lm) (inset) become visible. **(G)** Before introvert retraction, the musculature appears more developed. **(H)** Introvert retraction extends the circular and longitudinal musculature of the trunk, while short retractor muscles attach the introvert to the trunk. **(I)** The hatching larva exhibits a musculature similar to that of the late embryo, while **(J)** the first molt involves an increase in general complexity. **(E’-J’)** Schematic drawings depicting the basic muscular patterns. **A**, **C**, **E-J**, lateral views; **B**, vegetal view; and **D**, anterior view. All panels and drawings are oriented with the animal/anterior pole to the top. In **E**, ventral side to the left. The pairs of arrowheads in **C** and **E-J’** indicate the position of the introvert-trunk boundary. Drawings are not to scale. Scale bars in **E-H**, 50 μm; and **I** and **J**, 100 μm. an, anus; bp, blastopore; cm, circular muscles; em, endomesoderm; im, Introvert musculature; lrm, long retractor muscles; mo, mouth; pm, primary muscles; sc, scalids; srm, short retractor muscles; tm, trunk musculature.

After 6 days of embryogenesis, the basic organization of the priapulid gut emerges (Figure 2B, B’, Additional file 1: Figure S1A). The ectodermal mouth consists now of several tens of smaller ectodermal cells, and occupies an anterior terminal position, as observed in the adult. The endomesoderm is clearly segregated into endoderm and mesoderm: the gut appears as a straight tract connecting the anterior mouth to the posterior anus and is observed as a strongly actin-positive bundle, while the first signs of muscle differentiation become visible in the trunk and introvert (see also Figure 3F). At this stage, and based on whether cells belong to the external mono-layered ectoderm or not, the gut in *P. caudatus* consists of an anterior ectodermal mouth (foregut), an internal tract of at least partial endodermal origin, and a posterior ectodermal opening (anus) (Additional file 1: Figure S1B–E). The internal portion of the alimentary canal seems to be made of a limited and constant number of cells, being formed by three tetrads of cells and two pairs of cells serially arranged from anterior to posterior (Additional file 2: Video S1).

After the formation of the gut anlage, about days 7 to 8 of development, the introvert retracts and becomes sheathed in the trunk (Figure 2C,D,C’,D’). This is a key event during priapulid embryogenesis, as it results in the emergence of the larval/adult body plan [39]. Strikingly, when the introvert develops it is unfolded (Additional file 3: Figure S2). The animal-most ectoderm corresponds to the inner epidermis of the introvert, often called the oral or buccal cavity. At the most anterior region of the oral cavity, which in the embryo corresponds to the anterior region of the introvert-trunk boundary, the scalids (feeding teeth) develop ([34] and Figure 2B). The ectodermal indentation of the introvert-trunk boundary thus corresponds to the external epidermis of the introvert, the neck region (transition from the introvert and trunk), and the anterior epidermis of the trunk. During retraction, the initially extended inner gut (Figure 2C) is pulled down to the posterior end of the embryo (Figure 2D), as the introvert is incorporated inside the trunk, which also extends anteriorly during this process. As a result, the foregut, located at first at the anterior pole of the embryo, is internalized inside the embryo, and adopts a posterior position within the now folded introvert (Figure 2D, D’; Additional file 3: Figure S2). The posterior region of the embryo, and thus the anus, is not significantly affected by these major morphological rearrangements (Figure 2C,D,C’,D’). Additionally, introvert retraction is required for embryo hatching. The protrusion of the introvert eventually opens the hatching cap [35], allowing the hatching larva to escape.

A previous study of the external morphology of the hatching larva of *P. caudatus* reported the lack of mouth and anal openings in the larval cuticle [39]. Despite this absence, the hatching larva does show a fully developed digestive tract (Figure 2E,E’), similar to the one observed during embryonic development. No additional glands or attached organs are observed in close contact with the tube-like intestine. The first molting event, which results in the formation of the first lorica larva [39], involves a significant change in larval morphology and cell number (Figure 2F). The introvert and trunk grow in size and complexity, the internal portion of the alimentary canal is now formed by a greater number of cells, and the mouth and anal openings are present in the cuticle [39]. This observation suggests that the attainment of the mature digestive tract, as observed in the adult, is accomplished through successive molting events.

### Mesoderm development in *P. caudatus*

Segregation of endodermal and mesodermal precursors from a common endomesodermal germ layer is the first step in the development of their respective cell types and organs. During and immediately after gastrulation in *P. caudatus*, the endomesoderm shows no overt signs of segregation between endodermal and mesodermal populations (Figure 2A). However, there is expression of the endodermal marker *foxA* in the most animally located endomesodermal cells [34]. To identify the mesodermal precursors at this developmental stage, we analyzed the expression of the evolutionarily conserved mesodermal marker *twist* (*twi*) [40,41]. During gastrulation, *twi* transcripts are detected in the blastopore and the most vegetal endomesodermal cells, as well as in two lateral rows of internal cells (Figure 3A,B). Endoderm and mesoderm are thus likely distinct cellular populations already during gastrulation. As organogenesis proceeds through the introvertula stage, *twi* expression is detected in two broad rings of cells around the introvert and trunk (Figure 3C,D), which might correspond to the developing musculature (compare with phallacidin-positive muscles of the trunk and introvert in Figure 3F,G).

Differentiation of the mesoderm, and in particular of the surrounding visceral musculature, is essential for proper endoderm development in model organisms such as *D. melanogaster* and vertebrate embryos [4,28]. In *P. caudatus*, the organization of a recognizable gut tract by day 6 of development occurs simultaneously with the onset of muscle differentiation (Figure 3E,F). The first signs of this event are observed at the time of mouth formation, with the appearance of actin-positive circular fibers around the introvert-trunk boundary (Figure 3E). At the introvertula stage (Figure 3F), the body-wall musculature is obvious, with the development of circular muscles, mostly concentrated at the trunk level, and longitudinal muscles that connect the developing introvert with the trunk (inset Figure 3F). Before the retraction of the introvert (Figure 3G), the musculature appears further developed, in particular there are more muscle fibers at the introvert level. Introvert retraction, and thus the positioning of the digestive system in its final location, might be a muscle-controlled process, as is also the case during the protrusion and retraction of the adult introvert. As a consequence of the retraction of the introvert, the trunk musculature extends, and the circular packs of musculature and long retractor muscles become evident (Figure 3H). There are also shorter longitudinal retractor muscles connecting the posterior region of the introvert to the trunk. As observed with the digestive system, the musculature pattern observed in late embryos is conserved in the hatching larva (Figure 3I), and the number of muscle fibers increases after the first molting event (Figure 3J). Despite the fact that the adult priapulid gut is surrounded by a layer of longitudinal muscles that directly attaches to the basal lamina of the endoderm, our investigations point towards the absence of this musculature in priapulid embryos and first larval stages (see Figure 2E,F). The visceral musculature may thus develop in subsequent larval stages, in connection with the appearance of feeding behaviors [39] and a functional digestive system.

### Cell proliferation and cell migration during mouth development

**Figure 4:**
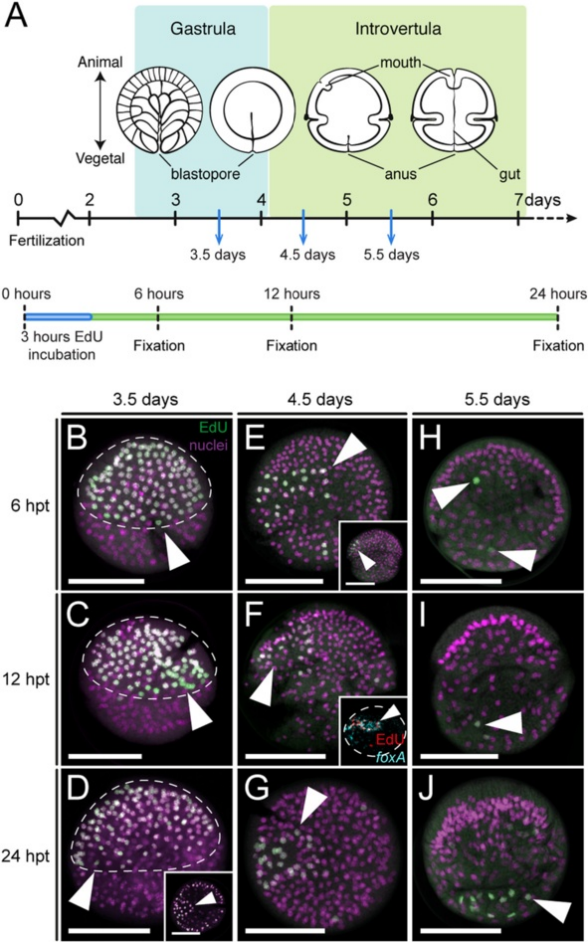
Cell proliferation during mouth development in *Priapulus caudatus*. **(A)** Schematic summary of the experimental setup to identify proliferative cells (by means of EdU incorporation) after 3.5, 4.5, and 5.5 days of development, and their position 6, 12, and 24 hours after the EdU pulse. Drawings are not to scale. **(B-J)** z projections of confocal stacks of embryos labeled for EdU-positive cells (green) and nuclei (magenta). **(B-D)** After gastrulation, cell proliferation is mostly concentrated in the animal hemisphere (white arrowheads, encircled by the dashed line), where the introvert and mouth (inset in **D**, white arrowhead) forms. **(E-G)** With the appearance of the ventral invagination that forms the mouth at 4.5 days of development, proliferation in the introvert becomes asymmetric (white arrowheads), on the side of mouth development, as observed by the co-localization of proliferative cells and cells expressing the mouth marker *foxA* (inset in **F**, white arrowhead; dashed line outlines the embryo). **(H-J)** Beyond 5.5 days of development, after mouth migration and the formation of the digestive tract, proliferative cells appear scattered throughout the introvert and trunk of the embryo (white arrowheads). In **B-D**, **F**, **H-J**, and inset in **E**, lateral view; and in **E**, **G**, inset in **D** and **F**, top view. In **D-J**, ventral to the left. Scale bars, 50 μm. hpt, hours post-treatment.

An anterior terminal position of the mouth has been proposed to be a plesiomorphic character in the Ecdysozoa [29,42]. To better understand the mechanisms governing the movement of the priapulid mouth from its ventral site of emergence to the most anterior tip of the body, we incubated embryos with the thymidine analog EdU to identify and track cells in the S-phase of the cell cycle. We treated embryos before mouth invagination (day 3.5 of development), at the point of ventral invagination (day 4.5), and when the mouth adopts an anterior terminal position (day 5.5), and fixed the treated embryos after 6, 12, and 24 hours (Figure 4A). With this set-up, we were able to detect active cycling cells at these points of development, and trace their position and the position of their daughter cells over the 24 hours following each respective labeling pulse.

Before mouth invagination, cell proliferation is mostly concentrated in the animal hemisphere of the embryo (Figure 4B), in the region that will form the introvert. This observation explains the greater number of nuclei observed in the introvert region using standard nuclear staining methods (for example, compare introvert and trunk regions in Figure 2B,C), and this region corresponds to the area of brain and proboscis formation. Localization of EdU-positive cells at 12 and 24 hours after the initial pulse demonstrated that labeled cells remained at the introvert region (Figure 4C,D), and that the mouth is formed by cells that originate in the animal hemisphere (inset in Figure 4D). Once the mouth invaginates on the ventral side of the embryo (Figure 4E, and inset), proliferation appears mostly concentrated on one side of the introvert, in a three- to four-cell-wide stripe that spans from the base of the introvert to almost the most anterior tip of the embryo. Individual proliferative cells are also observed in different parts of the introvert and trunk. Labeling for EdU-positive cells, together with cells expressing the oral marker *foxA* [34], showed that these populations are co-localized (Figure 4F, inset; Additional file 4: Figure S3), and indicates that the asymmetric proliferation observed in the introvert at this stage occurs ventrally, at the region of mouth formation and nervous system development [34]. At this stage, nuclei distribute more or less equally throughout the introvert ectoderm, except around the mouth and in the ventral midline where EdU-positive cells occur (Additional file 4: Figure S3), and ectodermal cells exhibit roughly the same size (see introvert region in Additional file 2: Video S1). Finally, cell proliferation decreases with the establishment of the basic body plan in the priapulid embryo after days 5.5 to 6 of development (Figure 4H-J), with only individual EdU-positive cells being observed in the introvert and trunk region after this time. Altogether, these results indicate that asymmetric cell proliferation is likely to be an important factor in the migration of the mouth from a ventral to an anterior terminal position, although they do not rule out that other factors also contribute to a certain extent. Additionally, the similar distribution of labeled cells at different time-points after a common EdU pulse suggests that cell migration is not a major force driving morphogenesis during *P. caudatus* development, as is also observed in the nematode *C. elegans* [21].

### Anteroposterior patterning of the digestive tract of *P. caudatus*

**Figure 5:**
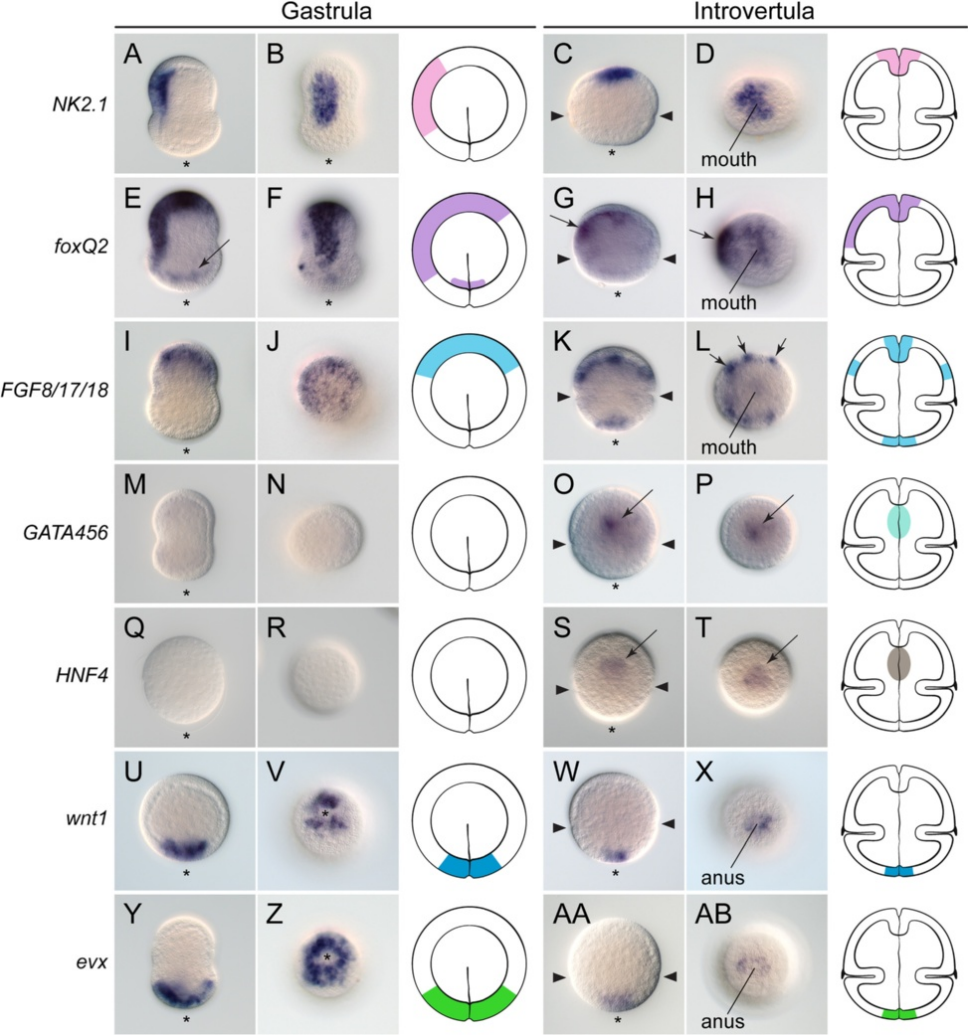
Anteroposterior patterning of the digestive system in *Priapulus caudatus* **.** Whole-mount *in situ* hybridization in priapulid embryos at the gastrula and introvertula stage. **(A-D)** The foregut marker *NK2.1* is expressed in the oral ectoderm at the gastrula stage, and in the mouth of the introvertula. **(E-H)** The apical ectoderm marker *foxQ2* is expressed in the animal and oral ectoderm of the gastrula, as well as around the blastopore (black arrow in **E**). In the introvertula, *foxQ2* is expressed in the mouth and developing nervous system (black arrows in **G**, **H**). **(I-L)** *FGF8/17/18* is expressed in the animal ectoderm during gastrulation, and in mouth, anus, and six ectodermal clusters (black arrows in **L**) of the introvert during organogenesis. **(M-P)** The midgut markers *GATA456* and **(Q-T)** *HNF4* are expressed in anterior midgut cells (black arrows in **O** and **S**) at the introvertula stage. **(U-X)** The posterior markers *wnt1* and **(Y-AB)** *evx* are expressed at the blastopore and vegetal pole during gastrulation, and in the posterior end of the trunk and anus at the introvertula stage. In all panels, the asterisk indicates the vegetal/posterior region. The pairs of arrowheads in **C**, **G**, **K**, **O**, **S**, **W**, and **AA** indicate the position of the introvert-trunk boundary. The schematic drawings of the gastrula and introvertula stage depict the reported expression domains. Drawings are not to scale. The slight elongation in the animal-vegetal axis of embryos at the gastrula stage is an artifact of fixation.

To characterize in greater detail the specification and formation of the different gut regions, we identified and studied the expression patterns of the anterior/foregut markers *NK2.1*, *foxQ2*, and *FGF8/17/18*; the midgut markers *GATA456* and *HNF4*; and the posterior/hindgut markers *wnt1* and *evx* (Figure 5). These markers complement our previous work describing the foregut markers *foxA*, *goosecoid* (*gsc*), and *orthodenticle* (*otx*), and hindgut markers *brachyury* (*bra*) and *caudal* (*cdx*) [34].

The oral ectoderm marker *NK2.1* [43] is expressed on one side of the gastrula, separate from the blastopore (Figure 5A,B). At the introvertula stage, *NK2.1* is expressed in the most apical region of the introvert, where the mouth is located (Figure 5C,D). *foxQ2* is a conserved marker of apical neural ectoderm [44,45], and in *C. elegans* and *D. melanogaster* it is also expressed in the foregut [46,47]. During gastrulation, *foxQ2* is expressed in the animal-most ectoderm, lateral ectoderm, and weakly in the ectoderm around the blastopore (Figure 5E,F). With the formation of the basic body plan at the introvertula stage, *foxQ2* becomes expressed around the mouth and on one side of the introvert, presumably the ventral side - which is also the case for the neural marker *otx* [34]. Finally, *FGF8/17/18* shows conserved expression at the mouth region in many studied bilaterians [48,49], and is detected in the animal hemisphere during gastrulation in *P. caudatus* (Figure 5I,J). At the introvertula stage, *FGF8/17/18* is expressed in the mouth and anus, as well as in six clusters of cells in the introvert, distributed in two bilaterally symmetrical rows of three clusters each (Figure 5K,L).

Orthologs of the *GATA456* subfamily and *HNF4* are evolutionarily conserved markers of the developing midgut [5]. Neither marker was detected at the blastula stage in *P. caudatus* (Figure 5M,N,Q,R), and their expression only became evident at the introvertula stage, in the inner cells right below the mouth, and thus presumably in the developing midgut (Figure 5O,P,S,T).

Finally, *wnt1* is a conserved marker of posterior regions across the Bilateria [50]. During gastrulation, *wnt1* is expressed vegetally, around the blastopore (Figure 5U,V), and this expression pattern remains at the introvertula stage, when *wnt1* is detected in the posterior tip of the trunk, and anus (Figure 5W,X). The homeobox-containing gene *evx* has been shown to play a conserved role in patterning the posterior regions of bilaterian embryos [51,52]. At the gastrula stage, *evx* is expressed broadly at the vegetal pole (Figure 5Y,Z), and as observed with *wnt1*, its expression becomes reduced to the posterior end of the trunk and anus at the introvertula stage (Figure 5AA,AB).

## Discussion

### Gut development in *P. caudatus*, and the ancestral state for the Ecdysozoa

**Figure 6:**
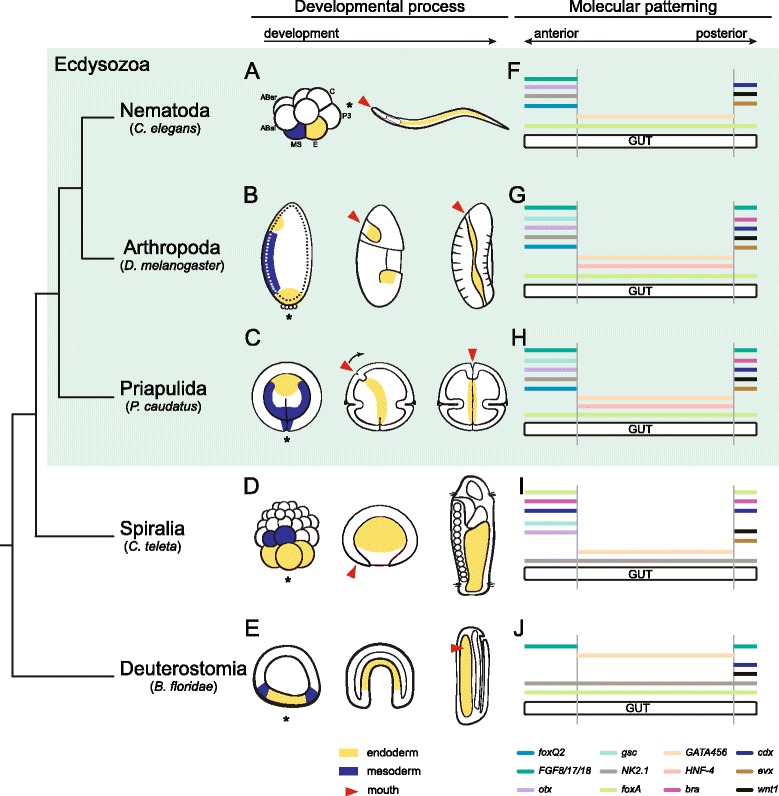
Evolution of endodermal differentiation in the Ecdysozoa. **(A**-**C)** Gut formation in *C. elegans*, *D. melanogaster*, and *P. caudatus*. See main text for details and references. Endoderm is in yellow and mesoderm in blue. Red arrowheads indicate the mouth. **(D)** In the Spiralia, the endoderm forms from the most vegetal blastomeres, which differentiate into the midgut. In *Capitella teleta*, the mesoderm mostly originates from the blastomeres 3c and 3d. The mouth forms ventrally, anterior to the closed blastopore. **(E)** In the Deuterostomia, the endoderm forms at the vegetal pole, and is internalized during gastrulation to form the midgut. The mesoderm develops from a ring of cells concentric to the endoderm (blue), and the mouth opens laterally in *Branchiostoma floridae*. **(F-J)** A comparison of the gut-related expression domains of *bra*, *cdx*, *eve*, *FGF8/17/18*, *foxA*, *foxQ2*, *GATA456* paralogs, *gsc*, *HNF4*, *NK2.1*, *otx*, and *wnt1* indicates the presence of a conserved gut patterning mechanism in the Ecdysozoa. Most of these genes are also related to gut development outside the Ecdysozoa, although their expression domains may appear in alternative gut regions or germ layers. In **F**, expression of the multiple *HNF4* paralogs of *C. elegans* is not depicted. In **F**, **G**, and **I**, expression of GATA456 paralogs is depicted under the same colored line. Mesoderm is only drawn at the stage of mesoderm specification. The asterisks indicate the vegetal/posterior pole. All embryos are oriented with the anterior/animal pole to the top and ventral to the left, except *C. elegans*, in which anterior is to the left and ventral to the bottom. Drawings are not to scale.

The most medial part of the digestive system usually originates from the endoderm, one of the two germ layers internalized during gastrulation in the Bilateria. In the Ecdysozoa, a vast variety of ontogenetic programs lead to the specification of the endoderm and its differentiation into a functional gut, mostly influenced by the particular ecological and developmental adaptations of each organism. The nematode *C. elegans* generates its whole midgut from the single E cell [21-24], located on the ventroposterior surface of the embryo (vegetal hemisphere) in the eight-cell-stage embryo (Figure 6A). The formation of the intestine from an early-specified founder cell seems to be common to most nematode lineages [53-57], and thus is likely the ancestral condition for this group. Nevertheless, nematomorphs, the sister group to nematodes [15], generate the endoderm from a vegetal population of blastomeres internalized during gastrulation [58,59]. Differing from nematodes and nematomorphs, the fruit fly *D. melanogaster* forms the midgut from two separate populations of endodermal cells located at the anterior and posterior pole of the embryo, respectively [26] (Figure 6B). This situation is observed in all winged insects (Pterygota) [60,61] but, in general, most other panarthropod embryos, such as those of myriapods, chelicerates, and onychophorans, form the endodermal cells at a defined point of the blastoderm [10,62-67]. These cells migrate over the yolk mass, and often phagocyte it, to eventually form the midgut. As an exception, crustaceans that develop via a hollow, radial blastula specify the endoderm from a small set of vegetal cells, sometimes even from just one cell [10,68-70]. Lastly, in the tardigrade *Thulinius stephaniae*, the intestine seems to originate from four founder blastomeres of different genealogical origins that are internalized after the primordial germ cells at the anterior pole [71]. Our data on the priapulid *P. caudatus*, a representative of the Scalidophora, show that its most medial region of the intestine likely forms from a single population of *foxA*-positive [34] endodermal cells that are internalized during gastrulation, and which occupy an anterior/animal position within the endomesoderm once gastrulation is completed (Figure 6C). The cell movements during gastrulation [35] suggest that these endodermal precursors are the first cells to internalize, and thus occupy the most vegetal region of the priapulid blastula. Nevertheless, detailed cell lineage studies and/or gene expression data at this developmental stage are required to confirm this hypothesis, and also, importantly, to address whether there is an additional contribution of midgut cells from the invaginating foregut ectoderm. Considering that holoblastic radial cleavage is plesiomorphic in the Ecdysozoa [36], and given the diversity of modes of endoderm specification within ecdysozoans, a single population of endodermal cells specified at the vegetal pole of the embryo is likely the ancestral condition in the Ecdysozoa - as is likely observed in priapulids, and also in nematomorphs, most holoblastic cleaving arthropods, and out-group representatives of the Spiralia and Deuterostomia (Figure 6D,E).

The organogenesis of a through gut from the primordial endodermal cells also varies among different ecdysozoan lineages. In *C. elegans*, the formation of the midgut occurs from a 16-cell primordium made of eight tiers of two cells each [18]. In this primordium, apical-basal cell polarization, lumen formation, and axial differentiation take place. The definitive midgut of the first larval stage strictly consists of 20 cells, and a similar olygocytose condition is observed in other members of the order Rhabditida and related taxa [54]. However, the majority of adult nematodes exhibit an intestine with hundreds or thousands of cells, which develops from a large midgut rudiment [54], and thus the situation observed in *C. elegans* is likely a derived condition. In most panarthropod embryos, the embryonic midgut is already made of multiple cells [10], as is also observed in *D. melanogaster* [25]. In *P. caudatus* embryos, the internal portion of the gut consists of 16 cells defining a tube and organized in three groups of four cells each and two posterior pairs of cells (Additional file 2: Video S1), a situation strikingly similar to the one described in *C. elegans*. However, successive rounds of molting seem to involve a general increase in the number of cells within the larval tissues and organs of *P. caudatus* (Figures 2 and 3), until reaching the polycytose situation of the intestine of adult priapulids. Notably, the priapulid hatching larva is non-feeding, as it lacks an oral and anal cuticular opening [39], and thus the olygocytose condition of the early post-embryonic intestine might be an adaptation to hatching with a yolk-rich immature gut. Taking everything into account, the development of a polycytose gut already during embryogenesis seems to be the ancestral condition in the Ecdysozoa.

### Mesoderm in *P. caudatus* and its relationship to endoderm development

The endoderm often develops in close association with the mesoderm - the internal germ layer that generates the musculature, blood system, excretory organs, and skeleton - and thus the endoderm and mesoderm frequently influence each other’s subsequent development [4,5]. In line with the variability in endoderm development observed in the Ecdysozoa, mesoderm segregation and differentiation also show great diversity [7,38]. In the nematode *C. elegans*, most larval mesoderm originates from the MS cell in the eight-cell stage embryo (Figure 6A), which is the sister cell of the endodermal E cell, both coming from the mother EMS cell in the four-cell stage embryo [21]. Ablation and cell culture studies have demonstrated that the E cell and its descendants have intrinsic properties to form polarized gut-like cells [18], and to pattern along the anteroposterior axis in a lineage-autonomous manner [72], although external factors and interactions with adjacent tissues, such as MS daughter cells [73] and the pharynx [74], are required for the proper definitive morphology of the digestive system. In early branching nematodes, there is no specification of the MS cell [53-55], and the formation of the embryonic midgut in relation to adjacent tissues has not been addressed. In the Nematomorpha, the exact origin of the mesoderm is not clear, although it appears as two lateral bands during gastrulation, surrounding the endoderm [58]. In the arthropod *D. melanogaster*, the mesoderm forms in the ventral region of the embryo, and is separated from the anterior and posterior midgut primordia by the foregut and hindgut ectoderm, respectively [26,60] (Figure 6B). The ingression of the mesoderm creates a ventral furrow, and its differentiation into the visceral mesoderm is essential for the proper development of the midgut cells and the formation of a through gut [28]. This situation seems to be common to most winged insects [60] and some apterygote (wingless) insects [75]. In other yolk-rich panarthropod embryos, mesoderm development is more variable [10] and can occur from a small posteroventral area of the blastoderm (for example, onychophorans [65,76]), or from individual cells delaminating from the blastoderm (for example, in some myriapods [63]). By contrast, in those marine crustaceans with holoblastic cleavage and hollow blastulae, the mesoderm originates from a small subset of vegetal blastomeres internalized with the endoderm during gastrulation, usually in the form of two lateral bands [10,69]. Finally, in the tardigrade *T. stephaniae*, the mesoderm originates from a variable number of blastomeres that internalize and proliferate as two bands along the left and right sides of the embryo, giving rise to the somites [71].

The expression of the mesodermal gene *twi* in *P. caudatus* at the gastrula stage (Figure 3) indicates that mesoderm originates from the most vegetal/posterior endomesodermal cells of the gastrula, and extends anteriorly as two lateral rows. According to a previous study [35], these two lateral mesodermal rows form through active proliferation, rather than by continuous ingression of cells through the blastopore. No visceral musculature is formed during embryonic development (Figures 2 and 3), although the presence of visceral mesodermal precursors within the population of *foxA*-positive gut cells remains a possibility. The visceral musculature thus probably appears in subsequent larval stages, given that this tissue is present in adult priapulids. However, the internal portion of the gut develops in close contact with the forming body wall musculature, and thus reciprocal interactions between endoderm and mesoderm cannot be completely excluded. Considering the different mechanisms observed in ecdysozoans, the ancestral mode of mesoderm formation is likely by the specification and internalization of mesodermal precursors along with the endodermal cells at the vegetal pole, and the formation of two lateral mesodermal bands through active proliferation that enclose the developing endoderm. Further functional investigations in *P. caudatus* and other ecdysozoan groups will be required to understand if the similarities in the interactions between the mesoderm and the endoderm observed in *D. melanogaster* and vertebrate embryos represent cases of convergence, or instead reflect ancestral developmental mechanisms.

### The question about the position of the mouth in the evolution of the Ecdysozoa

**Figure 7:**
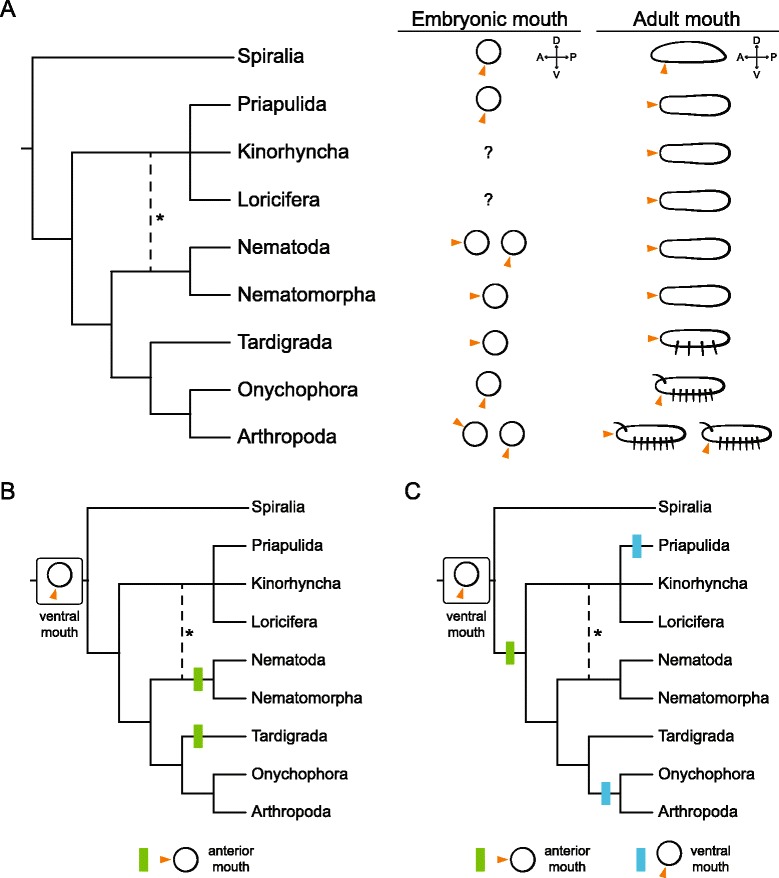
The evolution of the position of the mouth in the Ecdysozoa. **(A)** Diversity of mouth position in embryos and adults of the different ecdysozoan lineages. While in the Spiralia the mouth forms ventrally, and stays ventral in the adult, this situation is more variable in the Ecdysozoa. The mouth can form ventrally (priapulids, some nematodes, onychophorans, and most arthropods), at an anterior terminal position (most nematodes, nematomorphs, and tardigrades), or even on the dorsal site (in the pycnogonid arthropods). However, in the adults the mouth occupies an anterior terminal position, except in extant onychophorans and most arthropods (pycnogonids have a terminal mouth). While an anterior terminal mouth in the adult seems to be ancestral for the Ecdysozoa, the original embryonic position has been debated. **(B)** Evolutionary scenario of an ancestral ventral embryonic mouth. In this situation, independent modifications occurred in the Nematoida and Tardigrada that led to the late formation of the mouth at a terminal position. **(C)** Evolutionary scenario of an ancestral anterior embryonic mouth. In this situation, the embryonic mouth shifted to an anterior position at the base of the Ecdysozoa, and was secondarily reverted to a ventral site in priapulids and pan-arthropods, which is less parsimonious than assuming an ancestral ventral embryonic mouth (see **B**). For the sake of clarity, the ventral mouth of certain nematodes and the dorsal mouth of pycnogonids have not been considered in **B** or **C**. In **A**-**C**, the asterisk indicates the alternative branching of the Scalidophora (Priapulida, Kinorhyncha, Loricifera) together with the Nematoida to form what has been proposed as the Cycloneuralia clade. See main text for references. Drawings are not to scale.

Together with the midgut, the other two main regions of the digestive system in most bilaterian animals are the mouth (foregut) and the anus (hindgut). In the Ecdysozoa, the anus is of ectodermal origin, and forms at the posterior end, as in most other bilaterian groups, often ventrally and in relation to the site of gastrulation [34,77,78]. Differently from most other bilaterian animals, the mouth in adults of many ecdysozoan lineages is located at the most anterior tip of the body (terminal mouth), as observed in priapulids, kinorhynchs, loriciferans, nematodes, nematomorphs, tardigrades, and some arthropods (pycnogonids) (Figure 7A). Even stem group arthropods, such as fossil Cambrian lobopodians, exhibit an anterior terminal mouth [42]. This broad distribution has led to the current interpretation that the terminal mouth is an ancestral ecdysozoan character [29,42] that has been secondarily located to a ventral position in extant adult onychophorans and arthropods [79]. A convergent ventralization of the mouth opening is observed in strongylid nematodes that derive from an ancestor that possessed a terminal mouth [80]. Despite this seemingly uniform adult position, the ontogeny of the mouth varies among ecdysozoan lineages (Figure 7A). In *C. elegans* the mouth opens in a terminal anterior position ([19] and Figure 6A), independently from the site of invagination of the E-cell descendants. While this seems to be true for most other related nematodes, the mouth seems to form from a ventral blastopore in the marine Enoplea [53,54]. In nematomorphs and tardigrades [58,71], the mouth forms at the anterior pole. In the insect *D. melanogaster* (Figure 6B), as in most other panarthropod embryos, the mouth forms ventrally and remains there [10,26]. Quite unusually, in many pycnogonids, the only arthropod group with an anterior terminal mouth in the adult, the oral opening forms anterodorsally with respect to the chelifores (buccal appendages), and then moves ventrally to an anterior terminal position [36,81]. This dorsoventral movement relative to the first appendage pair also occurs in euchelicerates, although in this case the mouth ends up ventrally [10]. Our study shows that the mouth emerges ventrally in the priapulid *P. caudatus* ([34] and Figures 2, 3, 4, 5, 6C, and 7A), and then shifts anterodorsally towards its definitive anterior terminal position. Our time-course analysis of cell proliferation suggests that differential proliferation in the ventral ectoderm of the introvert might support this morphogenetic movement, although further studies will be required to test how alternative mechanisms, such as convergent-extension and cell intercalation [82-84], contribute to this process. Priapulid development thus delivers a clear example for how a strictly terminal mouth in the adult can originate from the ventral side of the embryo. Given that in other bilaterian lineages the adult mouth and its embryonic anlage is most often ventral (Figures 6D,F and 7A), the most parsimonious conclusion is that the ventral opening of the mouth observed in the embryos of the Priapulida, Onychophora, and most lineages of the Arthropoda is likely the ancestral developmental condition for the Ecdysozoa (Figure 7B,C). More derived nematodes, nematomorphs, and tardigrades, which open the mouth at the anterior terminal pole, would have thus lost the original ventral formation of the mouth, probably related to its relatively late opening in development [19,58,71]. In addition, onychophorans and most arthropods, which do open the mouth ventrally during embryogenesis, might have lost its subsequent shift to a terminal position and thus retained the ventral location in the adult. This modification could have been associated with the evolution of more elaborate head appendages and complex dorsal brains in the arthropod lineage, and thus our data can also contribute to explaining the long-standing problem of the evolutionary and ontogenetic origins of head structures in arthropods [85].

### Conserved molecular patterning of the *P. caudatus* gut

As discussed above, the ontogeny and adult architecture of the digestive system is highly variable between ecdysozoan lineages. This observation raises questions regarding the extent of differences in the molecular mechanisms underpinning gut development, and how these changes account for the manifest diversity of gut architectures. In the nematode *C. elegans*, endoderm specification is triggered by the maternally supplied bZIP/homeodomain gene *skn-1* (related to the *nrf2* gene of vertebrates, and the *cap’n’collar* gene of *D. melanogaster*), which is required for proper specification of the ventral EMS cell [86]. After the division of this cell, *skn-1* activates a cascade of redundant pairs of GATA factors (*med-1*, *med-2*, *end-1*, *end-3*, *elt-2*, *elt-4*, and *elt-7*) that will lead to the establishment of endodermal fate in the E cell [87], but not in the MS cell [88]. The Wnt pathway is also involved in this process [89], although this seems to be related to its general role in segregating cell fates along the anteroposterior axis [90]. Additionally, the transcription factor *pha-4*, an ortholog of the endodermal marker *foxA*, is expressed throughout the pharynx and midgut [91-93], and orthologs of *NK2.1*, *otx*, *FGF8/17/18*, and *foxQ2* (*C25A1.2*) are expressed and/or involved in pharynx development [19,46,94-96]. In nematodes, the nuclear hormone receptor family, and in particular the endodermal-related *HNF4*, has undergone extreme duplication [97], and many of the paralogs are expressed in different regions of the digestive system [98]. The posterior region and the hindgut, which consists of eight cells derived from the ABp blastomere [21], also show expression of *wnt1*, *evx*, T-box genes (not *bra*, which seems to be absent in *C. elegans*), *cdx*, and *pha-4* (*foxA*), among others [93,99-101] (see Figure 6F for a summary of expression data). In *D. melanogaster*, the specification of the midgut primordia is controlled by the terminal gap-gene *huckebein* (*hkb*), which controls endoderm specification at the amnioproctodeal invagination (posterior midgut), invagination of the anterior midgut, and specification of mesodermal precursors at the ventral furrow [102]. In *D. melanogaster*, *hkb* is a core component of the terminal patterning system, a development pathway involved in setting up the anterior and posterior ends of the embryo in hexapod arthropods [103,104]. However, its enrolment in this developmental pathway seems to be an evolutionary novelty, probably unique to *D. melanogaster* and closely related species, and its ancestral function was likely related to the nervous system [103]. The transcription factor *forkhead* (*foxA*); the GATA genes *serpent*, *grain*, and *dGATAe* (orthologs of the GATA456 subfamily); and the nuclear hormone receptor *HNF4* are subsequently required for proper midgut development in *D. melanogaster* [105-107]. Additionally, other genes such as *NK2.1*, *gsc*, *otx*, *foxQ2*, and *FGF8/17/18* are involved in the patterning of the head and foregut [47,49,108-110], and the genes *bra*, *cdx*, *wnt1*, *evx*, *FGF8/17/18*, and also *foxA* are required for the proper formation and patterning of the posterior region of the embryo [106,111-114] (Figure 6G). In the priapulid *P. caudatus*, the expression patterns of most of these genes exhibit significant similarities to the expression domains reported for *C. elegans* and *D. melanogaster* (Figure 6H). The F-box containing protein *foxA* is expressed in the foregut and inner gut [34], while a single *GATA456* gene and the *HNF4* ortholog are expressed in the anterior region of the internal alimentary canal. Together with *gsc* and *otx* [34], *NK2.1*, *foxQ2*, and *FGF8/17/18* are expressed in the foregut, while *wnt1*, *evx*, and also *FGF8/17/18* are detected in the ectodermal anus, as well as *bra* and *cdx* [34]. The expression of the endodermal midgut markers *GATA456* and *HNF4* is likely limited to the three most anterior tetrads of the internal gut, and the observation of the hindgut genes *foxA*, *bra*, and *cdx* [34] in the region corresponding to the two most posterior duplets could indicate that these regions correspond to the endodermal midgut and internal ectodermal hindgut of the priapulid embryo, respectively. More detailed cell lineage analyses will be required to confirm this hypothesis. Although functional data are still lacking in *P. caudatus*, the comparison of expression data with that of the nematode *C. elegans* and the insect *D. melanogaster* reveals important similarities between these lineages of ecdysozoans (Figure 6F-H), mostly during the stages in which the gut is patterned into the three main regions. Notably, the overall patterning of the digestive system appears to be more conserved between *P. caudatus* and *D. melanogaster*, although *P. caudatus* and *C. elegans* would be considered morphologically more similar [115,116]. *C. elegans* differs mostly by the absence (for example, *gsc* and *bra*) or expansion (*GATA456*, *HNF4*) of some of the studied genes, which might be related to its high rate of genome evolution [117]. Similarly, the differences in the earliest steps of endoderm development between *C. elegans* and *D. melanogaster* are probably due to their idiosyncratic early embryogenesis, as has also been shown in other bilaterian animals [6], and thus further work is needed to address the ancestral mechanism of endoderm specification for the Ecdysozoa. Nevertheless, our data on *P. caudatus* support the existence of a conserved molecular patterning program for the digestive system in the Ecdysozoa, despite the great differences in developmental modes and gut architectures.

The expression patterns of the above investigated genes in representative members of the ecdysozoan out-groups Spiralia (for example, the annelid *Capitella teleta*; Figure 6I) and Deuterostomia (for example, *Branchiostoma floridae*; Figure 6J) demonstrate that a similar system is also involved in gut regionalization outside the Ecdysozoa [48,51,52,118-128], although, in these organisms, the expression domains of particular genes often occur in, and extend to, different regions and germ layers. This observation, together with the similarities observed between *P. caudatus*, *C. elegans*, and *D. melanogaster*, strengthens the hypothesis of an ancestral molecular gut patterning system that is shared to a great extent between all the Ecdysozoa, despite morphological and developmental deviations being present in particular groups. Importantly, the molecular machinery that underlies early gut development in animals is much more similar than the developmental modes they undertake and the adult gut architectures they display (Figure 6). Therefore, the study of this common developmental toolkit alone cannot explain the vast morphological diversity of digestive tracts in animals. Differences in expression domains indicate that gene interactions and regulatory networks are probably variable, influenced by distinct developmental modes, early molecular/maternal inputs, and, most importantly, downstream effectors. Ultimately, the diversity of gut architectures also relies on molecular differences at more advanced stages of development. For instance, GATA factors activate effector genes required for intestinal cell differentiation in *C. elegans* [88,129,130], while triggering the epithelial-to-mesenchymal transition of the midgut primordia in the fly [107]. In a more general context, our study shows that the investigation of general patterning mechanisms between animals cannot lead to the prediction of a morphological outcome. A deeper understanding of the vast morphological diversity of animal forms can thus only be gained by broader taxon sampling and the consideration in developmental studies of the more terminal ontogenetic events that are ultimately responsible for the final morphological outcomes.

## Conclusion

Our comparative study of the development of *P. caudatus*, a representative of the sister group to all remaining ecdysozoans, shows that there are some primary features in the development of the digestive system that are likely to be ancestral for the Ecdysozoa, namely the formation of the endodermal midgut region from a single population of vegetal cells internalized during gastrulation, the ventral opening of the mouth and its subsequent shift to an anterior terminal position, and the development of the anus from the blastopore. Over evolutionary time, these characters have undergone great diversification and adaptation, as exemplified by the modes of gut development present in the two textbook invertebrate models, the nematode *C. elegans* and the fruit fly *D. melanogaster*. However, these extreme developmental divergences do not seem to be associated with a similar extent of molecular innovation in upstream patterning systems, as common transcriptional expression profiles are observed during the early stages of gut assembly among different ecdysozoan lineages. Our data not only shed light on the unexplored embryogenesis of the Priapulida and the evolution of the Ecdysozoa, but, importantly, also improve our understanding of the evolutionary changes that occurred in the lineages leading to *C. elegans* and *D. melanogaster*.

## Methods

### Animal collection, fertilization, and embryo fixation

Adult gravid specimens of *P. caudatus* were collected from Gullmarsfjorden (Fiskebäckskil, Sweden) in November in 2011, 2012 and 2013. Ovaries and testes were dissected, and kept in filtered deep seawater (FDSW). Oocytes were released by shaking the ovaries, and were fertilized with active diluted sperm from several males. Fertilized eggs were kept in petri dishes with FDSW at a constant temperature of 9°C, and washed daily with fresh FDSW to avoid bacterial and protozoan contamination. Embryos hatched 9 days after fertilization, and hatching larvae molted to the first lorica larvae 1 week thereafter, without any added food source. Before fixation, embryos were permeabilized with 0.05% thioglycolate, 0.01% pronase in FDSW for 45 min at 9°C. After three washes in FDSW, embryos were fixed in 4% paraformaldehyde in FDSW for 1 h at room temperature, followed by three washes in phosphate-buffered saline (PBS) with 0.1% Tween-20 (PTw). Hatching larvae and first lorica larvae were relaxed in 0.1% tricaine in FDSW for 30 s and fixed immediately in 4% paraformaldehyde in FDSW for 1 h at room temperature. Embryos and larvae fixed for immunohistochemical studies were stored in 0.1% sodium azide in PTw at 4°C. Samples fixed for gene expression studies were dehydrated in 50% methanol in PTw, washed once in 100% methanol, and stored in methanol at −20°C.

### Proliferation studies

Cell proliferation was observed by the incorporation of the thymidine analog EdU during DNA replication. Batches of embryos at days 3.5 (n = 18), 4.5 (n = 16), and 5.5 (n = 19) of development were incubated for 3 h in FDSW supplemented with 10 μM EdU. After this pulse, the medium was changed several times to remove any traces of EdU. Treated embryos were permeabilized and fixed as described above, 6 h, 12 h, and 24 h after the start of the EdU pulse, and stored in 0.1% sodium azide in PTw at 4°C. Fluorescent labeling of the incorporated EdU was performed as recommended by the Click-it EdU Alexa Fluor 488 imaging kit (Life Technologies, NY, USA), and nuclei were counterstained with 0.01 mg/mL propidium iodide.

### Phallacidin labeling

Embryos fixed and stored for immunohistochemical studies were washed several times in PBS before staining. Actin filaments and nuclei were labeled with 5 U/mL of Bodipy-FL phallacidin (Life Technologies, NY, USA) and 0.01 mg/mL propidium iodide (Sigma-Aldrich Chemie Gmbh Munich, Germany) in PBT (PBS, 0.2% TritonX-100, 0.1% bovine serum albumin) for 1 h at room temperature. Thereafter, embryos were washed in PBS for 1 h, dehydrated in a graded isopropanol series (70%, 85%, 95% in PBS, and twice in 100% for 30 to 60 s each) and cleared in Murray’s reagent (benzyl benzoate to benzyl alcohol, 2:1, v:v).

### Gene expression studies

A fragment of *NK2.1*, and the full-length sequences of *foxQ2*, *FGF8/17/18*, *GATA456*, *HNF4*, *wnt1*, *evx*, and *twi* [GenBank: KP013750–KP013757] were identified from RNAseq data. Protein alignments were constructed with MAFFT [131], and poorly aligned regions were removed with Gblocks [132]. RAxML [133] was used to infer gene orthologies (Additional file 5: Figure S4). Resulting trees were formatted with FigTree. Single colorimetric *in situ* hybridization was performed as described in [34]. Fluorescent *in situ* hybridization of *foxA* in EdU-treated embryos was performed following the regular colorimetric protocol up to antibody incubation, when samples were incubated overnight with an anti-DIG POD-conjugated antibody (Roche, Indianapolis, IN, USA) diluted 1:250 in blocking solution. After extensive washes, the signal was developed with a TSA-Cy3 kit (Perkin-Elmer, Waltham, MA, USA) following manufacturer’s recommendations. The TSA reaction was stopped in detergent solution (1% Triton X-100, 1% SDS, 0.5% sodium deoxycholate, 50 mM Tris pH 8, 150 mM NaCl) at 60°C, and embryos washed several times in PTw afterwards. Subsequent fluorescent labeling of the EdU incorporation in these embryos was performed as suggested by the EdU kit manufacturer (Life Technologies).

### Imaging

Fluorescence-stained embryos and larvae cleared in Murray’s reagent were scanned with a Leica SP5 confocal laser scanning microscope (Leica, Wetzlar, Germany). Embryos exhibiting representative expression patterns of the analyzed genes were cleared in 70% glycerol in PTw, and imaged with a Zeiss Axiocam HRc connected to a Zeiss Axioscope Ax10 using bright field Nomarski optics (Zeiss, Oberkochen, Germany). Images were analyzed in Fiji and Photoshop CS6 (Adobe), and figure plates made with Illustrator CS6 (Adobe).

## Additional files

Additional file 1: Figure S1. Cellular organization of the digestive system of *Priapulus caudatus*. z projections of confocal stacks of an embryo at day 6 of development stained with phallacidin (green) and propidium iodide (magenta). **(A)** Lateral view, with the digestive system fully developed: the mouth occupies an anterior terminal position, the midgut runs all along the embryo, and the anus opens posteriorly. The introvert-trunk boundary is well formed, and the scalids are visible. **(B)** Section of the introvert at the level of the mouth. Ectodermal cells of the mouth form a monostratified epithelium, with the apical side of the cells constricted and delimiting the lumen. Notice the connection of the mouth with the introvert ectoderm (white arrow), which corresponds to the neuroectoderm. **(C, D)** Sections through the midgut at the level of the introvert and trunk, respectively. Tiers of four cells delimit the central gut lumen. **(E)** Section of the trunk at the level of the ectodermal hindgut. In A, anterior to the top. The pair of arrowheads in A indicate the position of the introvert-trunk boundary, and the dashed lines the position of the transverse sections displayed on B-E). an, anus; dg, digestive system; lu, lumen; mc, muscles; mo, mouth; sc, scalids. Scale bars, 50 μm. Additional file 2:
**Video S1.** Structure of the embryonic digestive system. Video of a confocal stack of a 6-day-old embryo imaged along the anteroposterior axis. The morphology of the internal alimentary canal can be observed, as well as the morphology and distribution of ectodermal introvert cells. Additional file 3: Figure S2. Model for the retraction of the introvert. **(A)** The introvert-trunk boundary corresponds to the external introvert epidermis, neck, and part of the trunk epidermis, while the animal hemisphere ectoderm corresponds to the mouth and the oral cavity. **(B)** Before introvert retraction, the mouth moves to an anterior terminal position. **(C)** The retraction of the introvert starts when the introvert-trunk boundary unfolds, which pushes inwards the mouth and the oral cavity. This process is likely controlled by muscle contraction. **(D)** At the end of development, the introvert is retracted, the trunk completely covers the embryo, and the neck region lies at the top of the embryo. **(E)** When the hatching larva protrudes the introvert, the buccal cavity with the scalids and the mouth are projected anteriorly. The pairs of black arrowheads indicate the introvert-trunk boundary. All embryonic stages and the hatching larva are oriented with the anterior to the top. Drawings are not to scale. an, anus; dg, digestive system; mo, mouth; sc, scalids. Additional file 4: Figure S3. Position of EdU-positive cells during mouth development. **(A’-A”’)** z projections of confocal stacks of an embryo after 4.5 days of development labeled for EdU-positive cells (green) and *foxA*-positive expressing cells (magenta). The mouth marker *foxA* is expressed ventrally at the mouth ectoderm [34], and co-localizes with the EdU proliferative cells, which are thus present around the mouth region (mo) and ventral side of the introvert. **(B)** z projections of a confocal stacks of an embryo at a similar developmental time point, showing that nuclei distribute more or less uniformly throughout the introvert ectoderm, but slightly more densely packed in the ventral midline and mouth area (delimited by the dotted line), which corresponds to the EdU-positive region in A. A’-B, anterior views. Scale bars, 25 μm. Additional file 5: Figure S4. Analyses of gene orthology. **(A-H)** Maximum likelihood phylogenetic trees of *twi*, *NK2.1*, *foxQ2*, *FGF8/17/18*, *GATA456*, *HNF4*, *wnt1*, and *evx*. Replicate bootstrap values were calculated with the autoMRE option in RAxML. *P. caudatus* sequences are highlighted in red. Models of protein evolution used for each tree: *twi*, JTT; *NK2.1*, RtREV; *foxQ2*, WAG; *FGF8/17/18*, WAG + F; *GATA456*, JTT; *HNF4*, LG; *wnt1*, WAG; and *evx*, LG.
