# Supplementary figures and images for: The study of Priapulus caudatus reveals conserved molecular patterning underlying different gut morphogenesis in the Ecdysozoa
Source: BMC Biol. 2015 Apr 21;13:29. doi: 10.1186/s12915-015-0139-z (PMC4434581; doi:10.1186/s12915-015-0139-z)

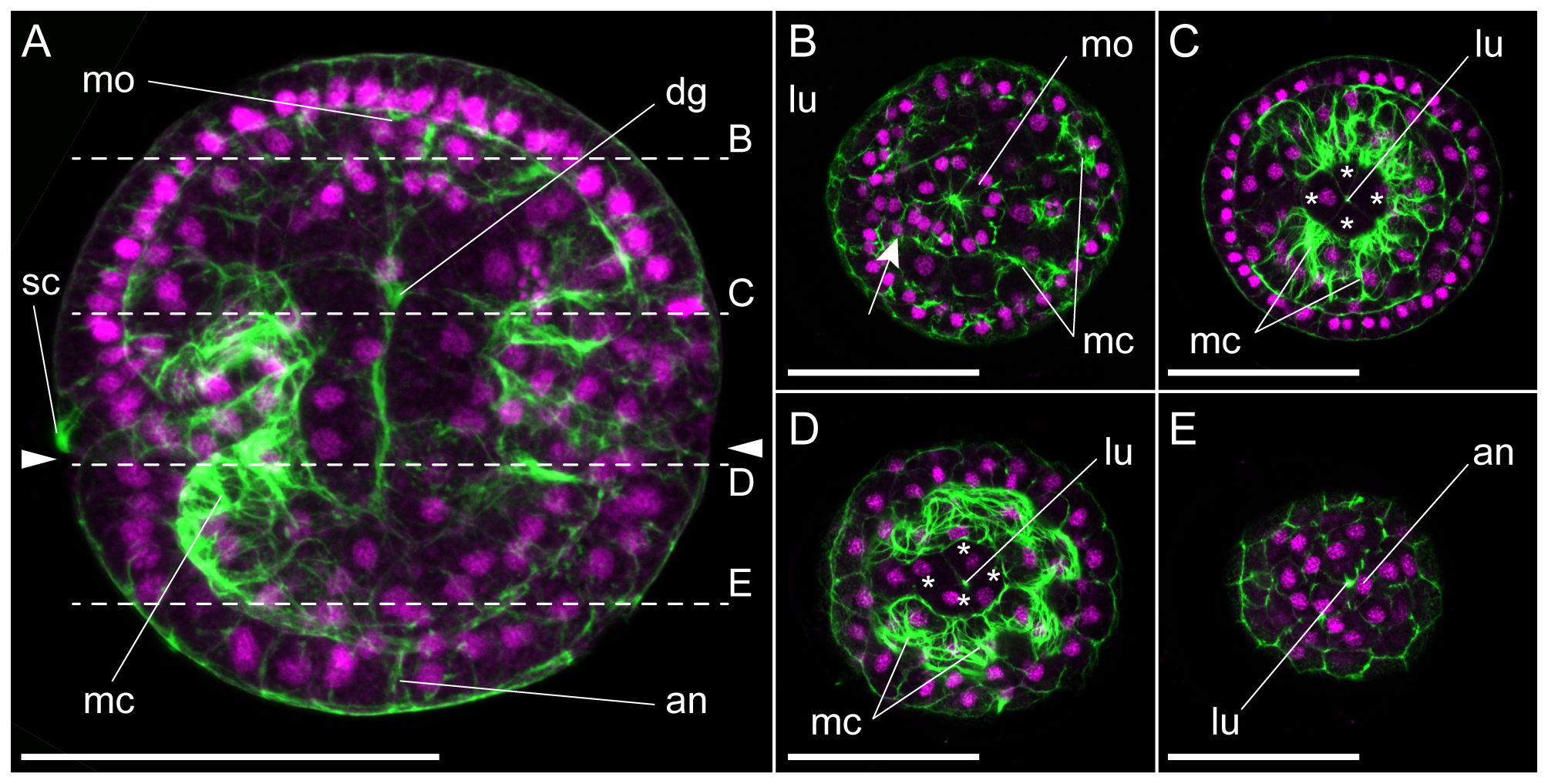

Supplement: Additional file 1: Figure S1. — Cellular organization of the digestive system of Priapulus caudatus. z projections of confocal stacks of an embryo at day 6 of development stained with phallacidin (green) and propidium iodide (magenta). (A) Lateral view, with the digestive system fully developed: the mouth occupies an anterior terminal position, the midgut runs all along the embryo, and the anus opens posteriorly. The introvert-trunk boundary is well formed, and the scalids are visible. (B) Section of the introvert at the level of the mouth. Ectodermal cells of the mouth form a monostratified epithelium, with the apical side of the cells constricted and delimiting the lumen. Notice the connection of the mouth with the introvert ectoderm (white arrow), which corresponds to the neuroectoderm. (C, D) Sections through the midgut at the level of the introvert and trunk, respectively. Tiers of four cells delimit the central gut lumen. (E) Section of the trunk at the level of the ectodermal hindgut. In A, anterior to the top. The pair of arrowheads in A indicate the position of the introvert-trunk boundary, and the dashed lines the position of the transverse sections displayed on B-E). an, anus; dg, digestive system; lu, lumen; mc, muscles; mo, mouth; sc, scalids. Scale bars, 50 μm. [file 12915_2015_139_MOESM1_ESM.tif]

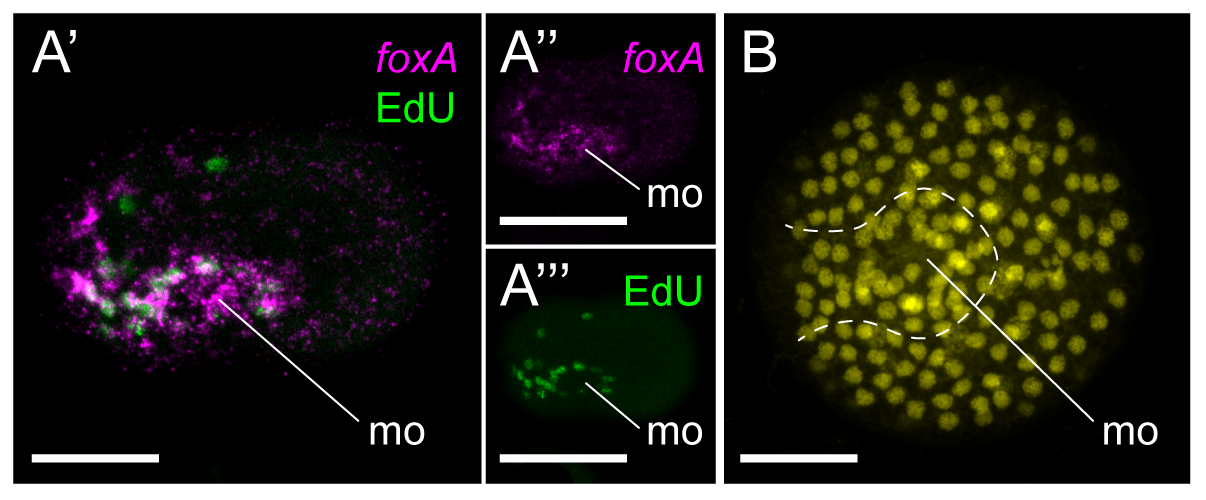

Supplement: Additional file 4: Figure S3. — Position of EdU-positive cells during mouth development. (A’-A”’) z projections of confocal stacks of an embryo after 4.5 days of development labeled for EdU-positive cells (green) and foxA-positive expressing cells (magenta). The mouth marker foxA is expressed ventrally at the mouth ectoderm [34], and co-localizes with the EdU proliferative cells, which are thus present around the mouth region (mo) and ventral side of the introvert. (B) z projections of a confocal stacks of an embryo at a similar developmental time point, showing that nuclei distribute more or less uniformly throughout the introvert ectoderm, but slightly more densely packed in the ventral midline and mouth area (delimited by the dotted line), which corresponds to the EdU-positive region in A. A’-B, anterior views. Scale bars, 25 μm. [file 12915_2015_139_MOESM4_ESM.tif]

Figure S4

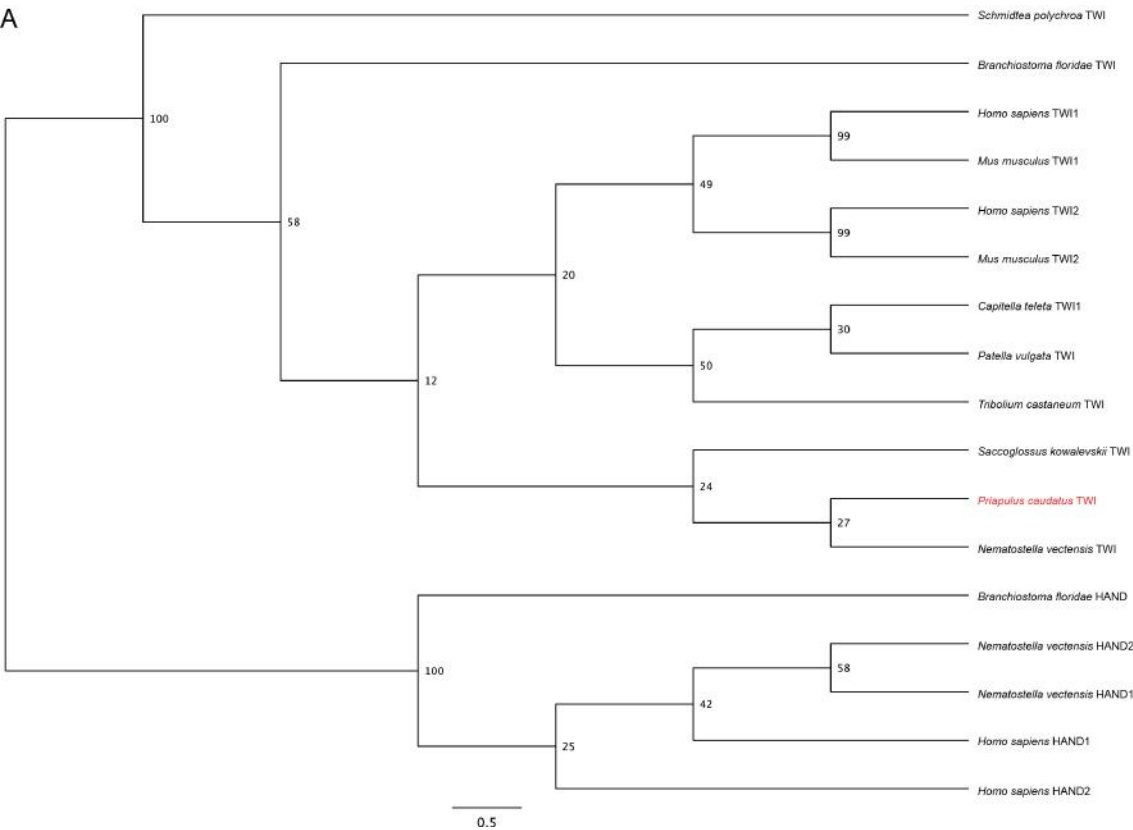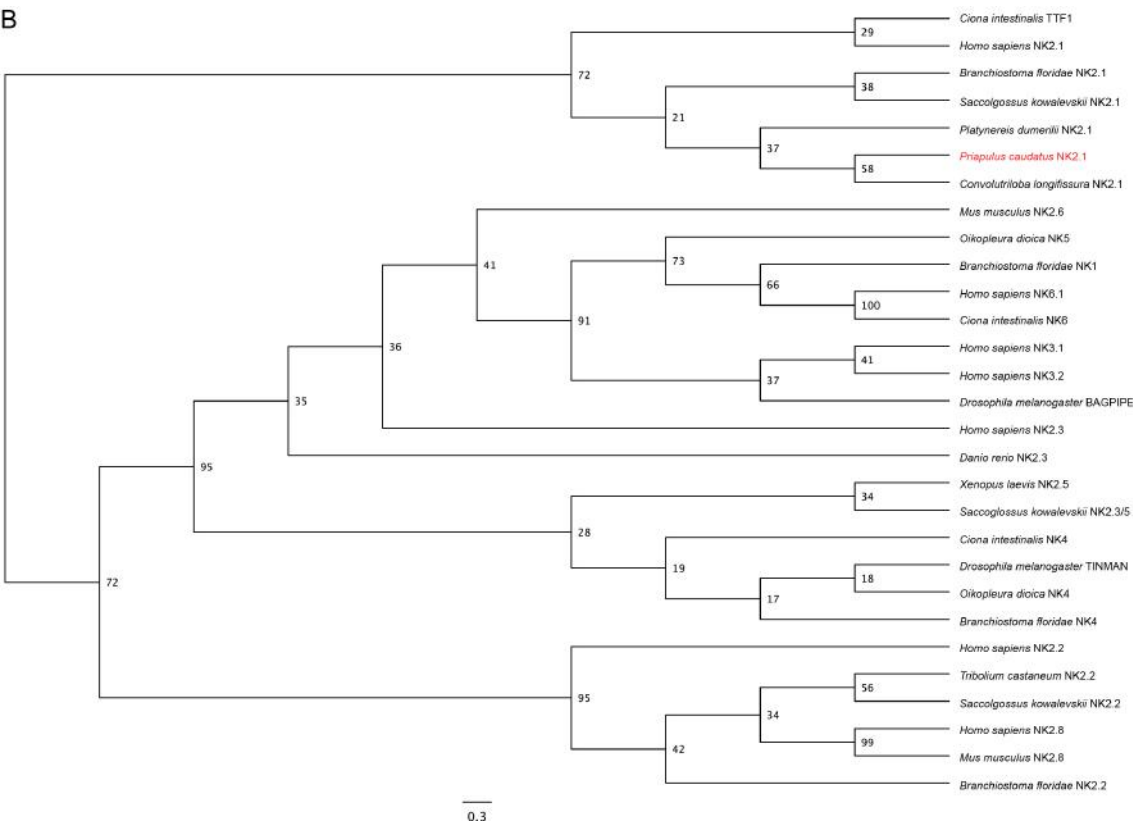

C

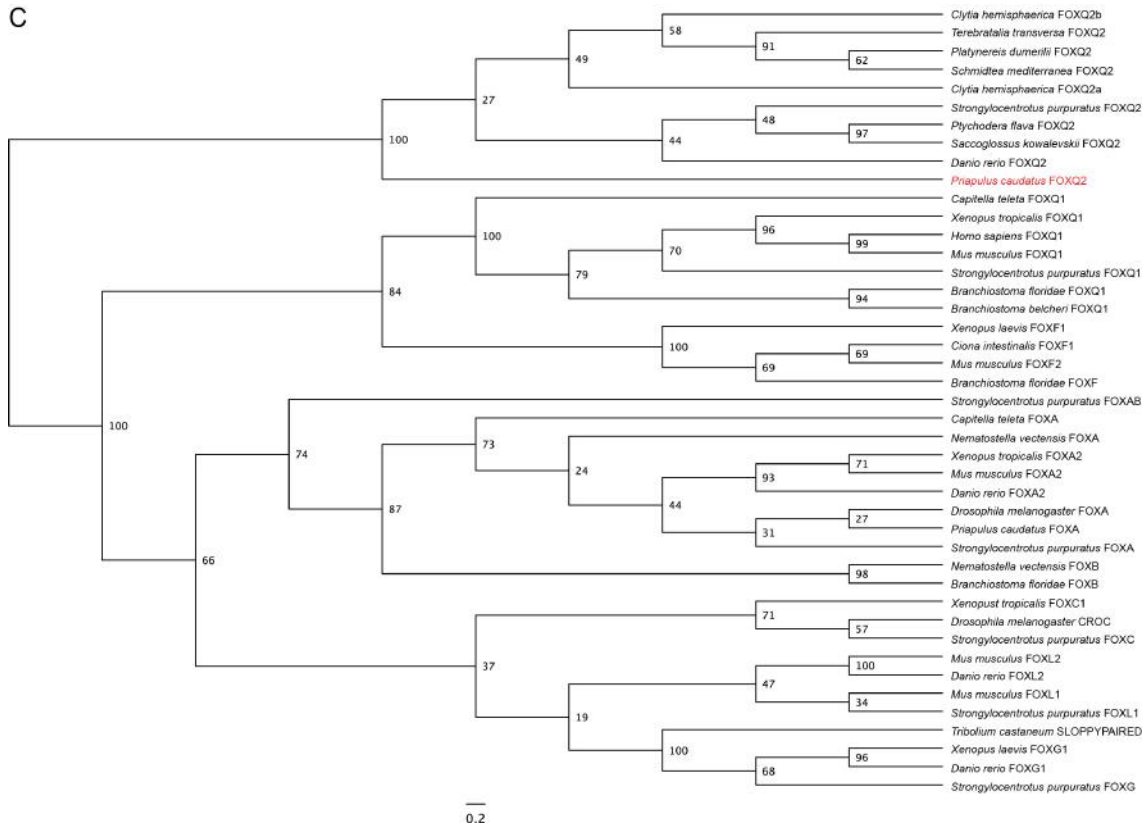

D

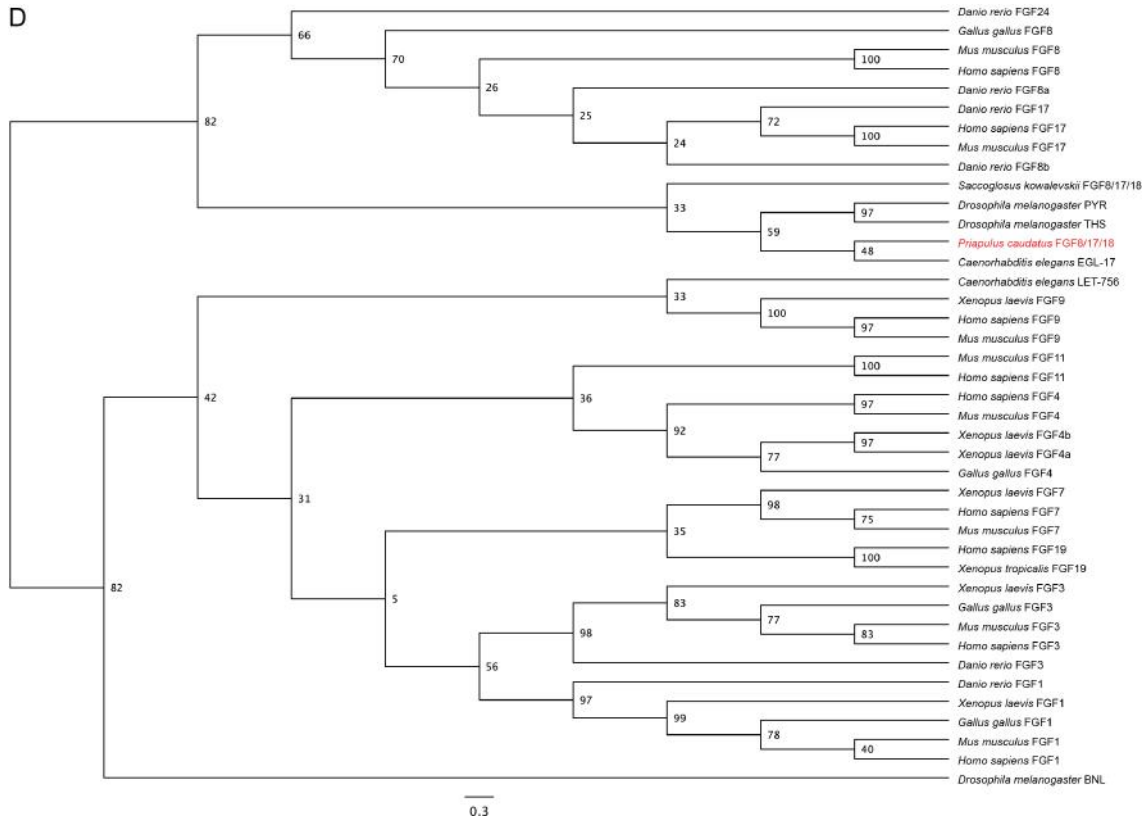

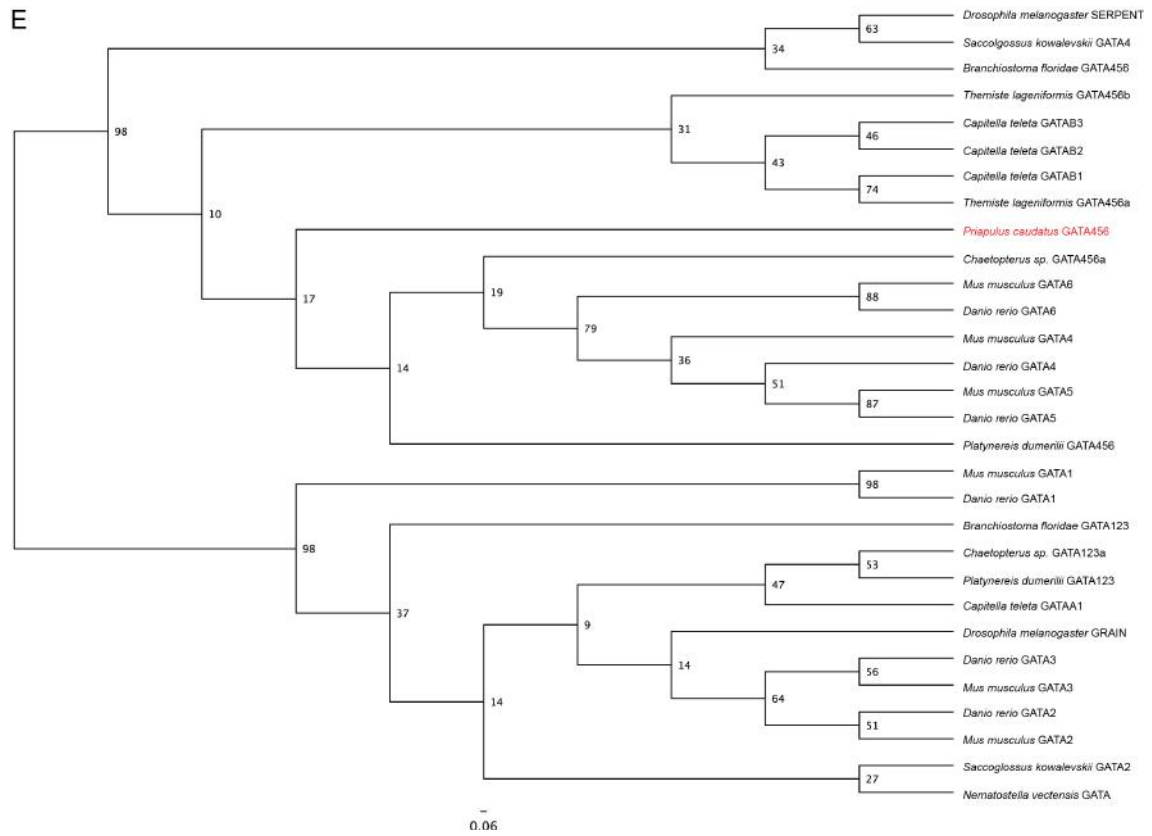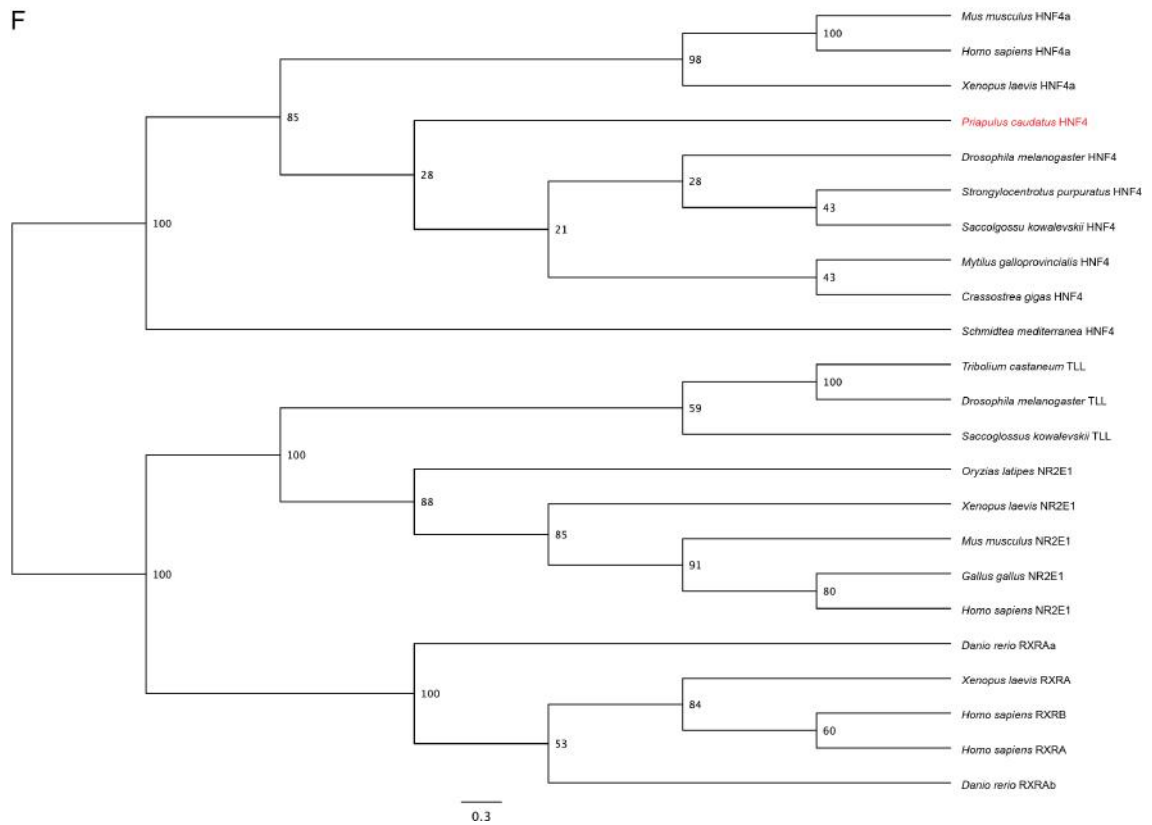

G

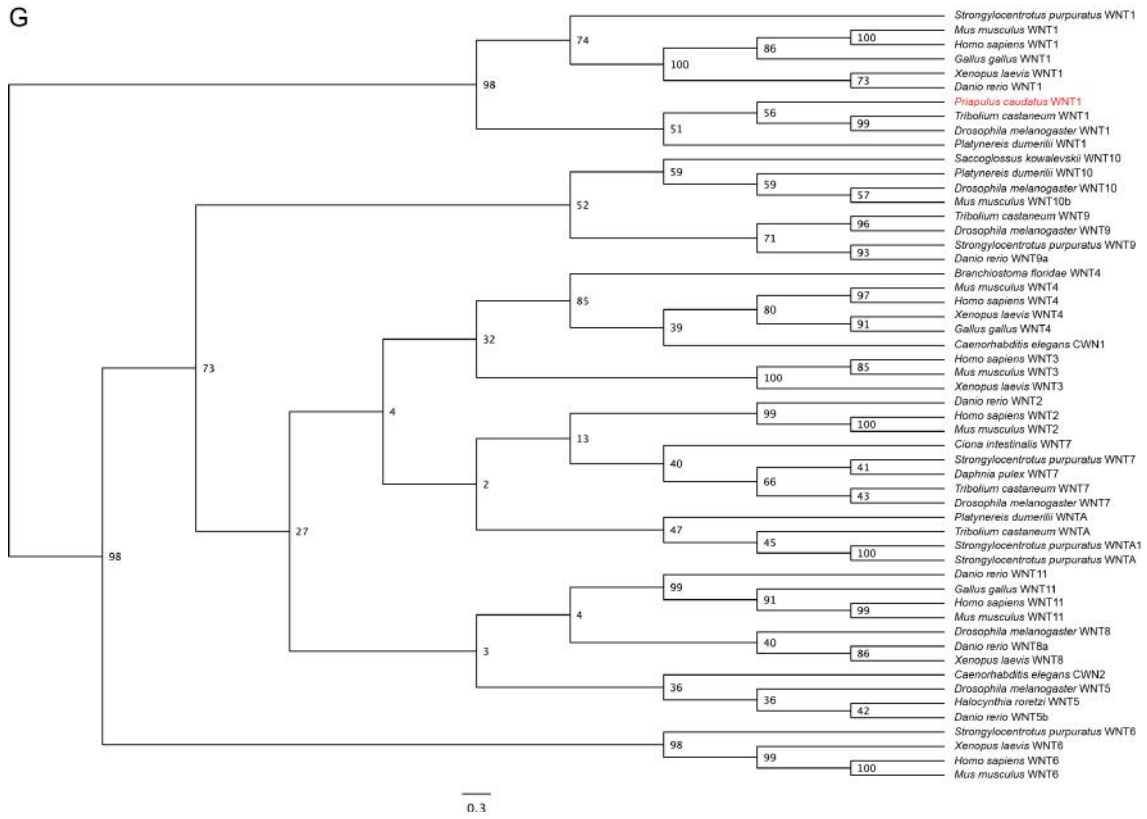

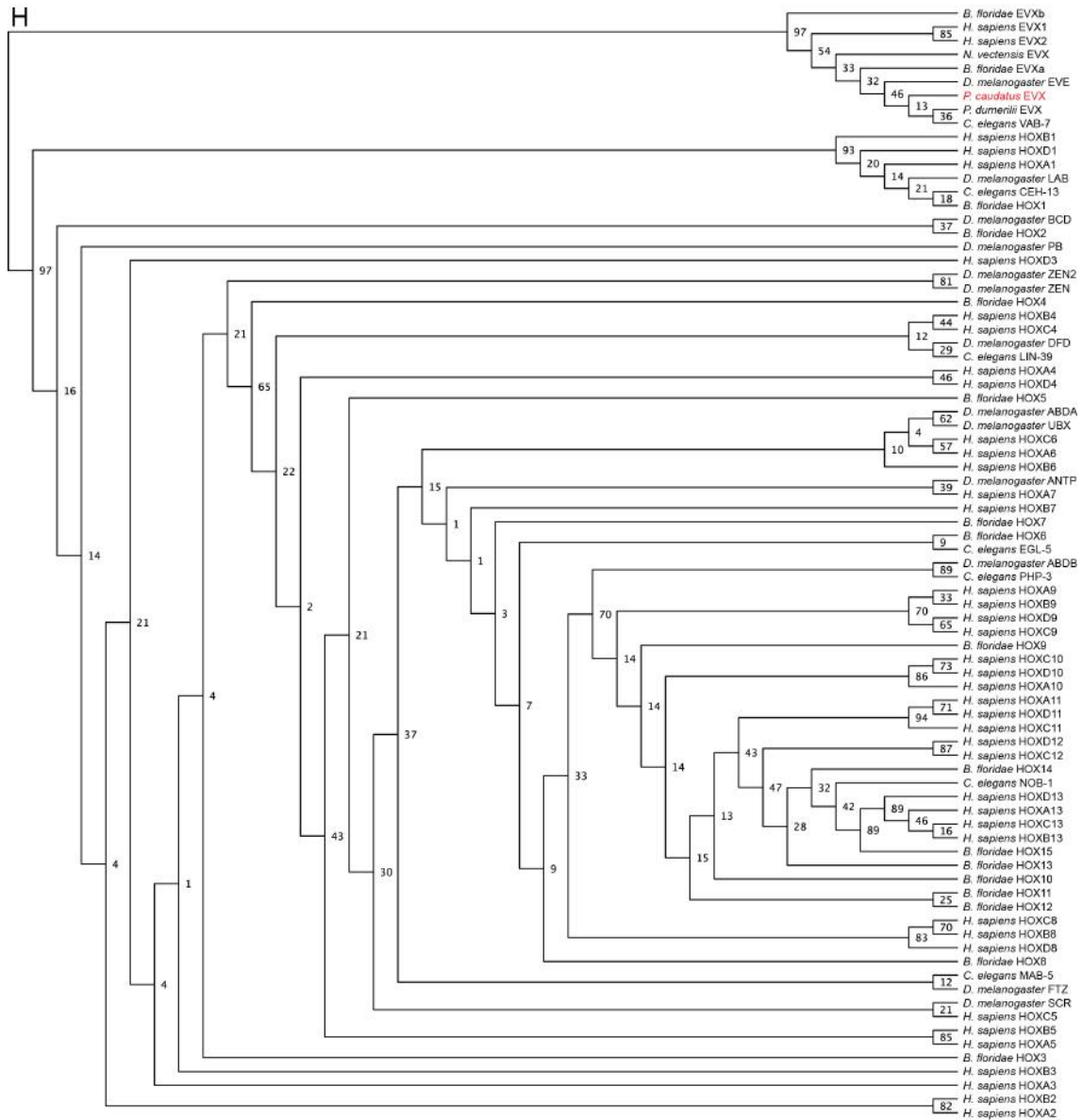

Supplement: Additional file 5: Figure S4. — Analyses of gene orthology. (A-H) Maximum likelihood phylogenetic trees of twi, NK2.1, foxQ2, FGF8/17/18, GATA456, HNF4, wnt1, and evx. Replicate bootstrap values were calculated with the autoMRE option in RAxML. P. caudatus sequences are highlighted in red. Models of protein evolution used for each tree: twi, JTT; NK2.1, RtREV; foxQ2, WAG; FGF8/17/18, WAG + F; GATA456, JTT; HNF4, LG; wnt1, WAG; and evx, LG. [file 12915_2015_139_MOESM5_ESM.pdf]
